# The Role of Transthyretin in Oligodendrocyte Development

**DOI:** 10.1038/s41598-020-60699-8

**Published:** 2020-03-06

**Authors:** Bandar Alshehri, Maurice Pagnin, Jae Young Lee, Steven Petratos, Samantha J. Richardson

**Affiliations:** 1 School of Health and Biomedical Sciences, RMIT University, Bundoora, Victoria, 3083 Australia; 2 Faculty of Applied Medical Sciences, Najran University, Najran, Saudi Arabia; 3 Department of Neuroscience, Central Clinical School, Monash University, Prahran, Victoria, 3004 Australia; 4 ToolGen, Inc., Seoul, 08501 Korea; 5 School of Science, RMIT University, Bundoora, Victoria, 3083 Australia

**Keywords:** Oligodendrocyte, Myelin biology and repair

## Abstract

Transthyretin (TTR) is a protein that binds and distributes thyroid hormones (THs) in blood and cerebrospinal fluid. Previously, two reports identified TTR null mice as hypothyroid in the central nervous system (CNS). This prompted our investigations into developmentally regulated TH-dependent processes in brains of wildtype and TTR null mice. Despite logical expectations of a hypomyelinating phenotype in the CNS of TTR null mice, we observed a hypermyelination phenotype, synchronous with an increase in the density of oligodendrocytes in the corpus callosum and anterior commissure of TTR null mice during postnatal development. Furthermore, absence of TTR enhanced proliferation and migration of OPCs with decreased apoptosis. Neural stem cells (NSCs) isolated from the subventricular zone of TTR null mice at P21 revealed that the absence of TTR promoted NSC differentiation toward a glial lineage. Importantly, we identified TTR synthesis in OPCs, suggestive of an alternate biological function in these cells that may extend beyond an extracellular TH-distributor protein. The hypermyelination mechanism may involve increased pAKT (involved in oligodendrocyte maturation) in TTR null mice. Elucidating the regulatory role of TTR in NSC and OPC biology could lead to potential therapeutic strategies for the treatment of acquired demyelinating diseases.

## Introduction

Transthyretin (TTR) is a homo-tetrameric protein with a molecular mass of ~55 kDa^1^. TTR synthesis in the central nervous system (CNS) was first described in the brain^2^. Soon thereafter, TTR mRNA was reported to be only in the choroid plexus of the brain^3^, with specific expression in the choroid plexus epithelial cells^4^. The main protein synthesized and secreted by choroid plexus epithelial cells of mammals, birds and reptiles is TTR^5^. This TTR is secreted into the cerebrospinal fluid (CSF)^6^ and is involved in moving thyroxine (T4) from the blood via the choroid plexus into the (CSF) and throughout the brain parenchyma^6–9^.

Despite its established role as a T4 distributor protein throughout the CNS, it has been suggested that TTR can be synthesized in human and rodent dorsal root ganglia (DRG)^10^. However, the validity of these data was questioned because the *TTR* mRNA may have been from contaminating meningeal cells^11^. Thus, laser capture micro-dissection was implemented to investigate specific DRG expression of the *TTR* gene without contamination from the surrounding meninges^12^. This technique not only confirmed that the *TTR* gene is expressed in the DRG but also in the sciatic nerve of mice. The level of detected *TTR* mRNA in the mouse sciatic nerve was one-fifth of the *TTR* mRNA in the DRG^12^, thereby proposing an alternate role for TTR in the nervous system, albeit in the periphery.

Consolidating an alternate hypothesis for the function of TTR, was the recent discovery that primary cultured Schwann cells isolated from mouse sciatic nerves expressed *TTR* mRNA^12^. It was also observed that a significant decrease in the level of *TTR* mRNA in rat Schwann cell cultures occurred after differentiating these cells in culture through the provision of forskolin^12^, possibly suggesting that TTR may play a role in these cells during their immaturity. Beside immature peripheral glia showing a transient TTR expression profile, recent data obtained from a secretory molecular analysis identified that astrocytes and transit-amplifying cells (type-C cells) in the subventricular zone (SVZ) of the CNS may synthesize TTR^13^. The function of TTR synthesis *de novo* in the CNS however, may well be related to its known function as a T4 distributor protein. Until now, a function for TTR synthesized by neural cells is yet to be elucidated and so an alternate biological function outside of its T4 distributor role remains a plausible hypothesis.

The generation of the TTR null mouse^14^ that was viable and fertile raised questions about the importance of choroid plexus derived TTR in facilitating the passage of T4 from the blood into the CSF (for a recent review see^15^). TTR null mice consistently exhibit reduced levels of total T4 and T3, retinol and retinol-binding protein in their peripheral blood^14,16,17^. The direct effects of these deficits in the CNS are yet to be clearly elucidated, however, we have previously reported that TTR null mice exhibit reduced apoptosis of NSCs in the SVZ compared with those in wild type mice^18^. Additionally, it has been recently suggested that temporary inactivation of T3 in the SVZ creates favourable conditions for NSCs to commit to an oligodendroglial lineage rather than a neuronal fate^19^. The proportion of apoptotic cells in the SVZ of TTR null mice was equivalent to that seen in the SVZ of hypothyroid wild type mice^18^, supporting a relationship between TH regulation and cell cycle events within germinal centres of the brain^20^. Furthermore, we reported that TTR null mice had delayed CNS development as indicated by elevated protein concentration in the CSF until at least P14^17^. These data prompted our investigations into the well-documented TH-dependent process that occurs during brain development and regulated from the SVZ: myelination of the commissural fibres within the CNS^21^.

A hallmark of hypothyroidism is reduced myelination of axons of the corpus callosum and the anterior commissure^22^. Therefore, we expected a hypomyelination phenotype during postnatal development of TTR null mice. Surprisingly, we found a hypermyelination phenotype, accompanied by an increase in the number of adenomatous polyposis coli clone CC1-positive (CC1-positive) oligodendrocytes, accelerated maturation, proliferation and migration of (oligodendrocyte precursor cells) and a decreased rate of apoptosis of OPCs in TTR null mice. Lack of TTR promoted neural stem cell (NSC) differentiation into glial cell linages. Optic nerves from TTR null mice had greater levels of phosphorylated protein kinase B (also known as AKT) compared to those from wild type mice, suggesting a mechanism for hypermyelination. Finally, we identified TTR synthesis by OPCs. We correlated the lack of TTR with the hypermyelination phenotype in TTR null mice.

## Materials and Methods

### Animals

Mice of five different age groups were used: E18, P7, P14, P21 and 12 weeks. All animal experiments were performed with the approval of RMIT University Animal Ethics Committee (AEC# 1209) and conformed to the Australian National Health and Medical Research Council guidelines. All mice were housed in individually ventilated cages in the breeding facility of the RMIT University Animal Facility (RMIT University, Victoria, Australia) with *ad libitum* access to rat chow (iodine 0.5 mg/kg; Specialty Feeds Co. Western Australia) and tap water at room temperature (22 ± 1 °C) with a 12:12-h light-dark regimen.

Mice (wild type and TTR null) were on a C57Bl6 background and mice of both sexes were used (sex of each mouse recorded). Housing was in Allentown IVC (individually vented caging systems) cages. The number of cage companions: Breeders were 2 mothers plus nursing pups; stock mice were housed according to their sex and litter (up to 4 per box). Environmental enrichment: bedding was Aspen bedding fine grade; nesting material: Aspen nesting material and Puracrinkle nests; food: specialty feeds – irradiated rat and mouse pellets, gnaw sticks, mouse domes, autoclaved toys. All procedures on animals were done in the RMIT University Animal Facility.

### Human oligodendrocyte progenitor cells

Human oligodendrocyte precursor cells (hOPCs) derived from NIH approved H9 human embryonic stem cells (hESCs) were purchased from Merck Millipore (CS204496). For proliferation, hOPCs were cultured in Human OPC Expansion Complete Medium supplemented with recombinant human bFGF (50 µg/ml), OPC Expansion Supplement A (PDGF-AA) 2500x, OPC Expansion Supplement B (NT3) 2500x and 1% (v/v) streptomycin-antimycotic. Culture flasks were coated with Geltrex (Invitrogen, 12760013). Cells were proliferated for 3 weeks then analytical studies were performed.

For differentiation, hOPCs were cultured in Human OPC Spontaneous Differentiation Complete Medium supplemented with Neural Supplement 1 (50x), (without PDGF-AA or basic FGF growth factors). Culture flasks were coated with 100 μg/ml poly-L-ornithine and 10 μg/ml laminin. Cells were differentiated for two weeks and then analytical studies were performed.

### Isolation of neural stem cells from the subventricular zone of mouse brains

Primary (NSCs) were isolated from the (SVZ) of 21-day-old TTR null and wild type mouse brains. The SVZ areas were dissected using a protocol described previously^23^. Next, NSCs were isolated using a Neural Tissue Dissociation kit (Miltenyi Biotec, 130-092-628), according to the manufacturer’s instructions. Isolated NSCs were seeded in a coated T25 flask for monolayer  NSC cultures and in an uncoated 6-well plate for neurosphere cultures. The flasks were incubated at 37 °C and 5% CO_2_ for one week and the culture medium was changed every two days.

### Neural stem cell differentiation assay

In order to investigate the multipotency of NSCs in neural stem cell NSC-progeny, a monolayer of cells was established for isolated NSCs. The NSCs were seeded in 6-well clear tissue culture plates coated with 10 μg/ml poly-L-ornithine and 10 μg/ml laminin, with a cell density of 5 × 10^5^ per well. Proliferation Complete Medium was added to the cells and incubated for 14 hours to allow the cells to attach. The next day, 80% of the proliferation medium was removed and replaced with fresh differentiation medium. Three changes of differentiation medium were used to direct the NSCs to specific cell types (Table 1). The differentiation medium was changed every two to three days over a period of three weeks. At the end of that period, the cells were harvested, fixed, and flow cytometry was performed to quantify the number of differentiated cells (see below).

**Table 1:** Media used for NSC differentiation assays.

Medium type	Component	Company	Final concentration
NSC/OPC proliferation complete medium	KnockOut™ D-MEM/F-12	Invitrogen	1X
	StemPro® Neural Supplement	Invitrogen	2%
	GlutaMAX™-I Supplement	Invitrogen	2 mM
	bFGF (100 μg/ml stock)	Invitrogen	20 ng/ml
	EGF (100 μg/ml stock)	Invitrogen	20 ng/ml
	Antibiotic-Antimycotic	Invitrogen	1X
NSC differentiation medium	Neurobasal® Medium	Invitrogen	1X
	GlutaMAX™-I Supplement	Invitrogen	2 mM
	B-27® Serum-Free Supplement	Invitrogen	2%
	Antibiotic-Antimycotic	Invitrogen	1X
Astrocyte differentiation medium	D-MEM	Invitrogen	1X
	N-2 Supplement	Invitrogen	1%
	GlutaMAX™-I Supplement	Invitrogen	2 mM
	FBS	Invitrogen	1X
	Antibiotic-Antimycotic	Invitrogen	1X
Oligodendrocyte differentiation medium	Neurobasal® Medium	Invitrogen	1X
	GlutaMAX™-I Supplement	Invitrogen	2 mM
	B-27® Serum-Free Supplement	Invitrogen	2%
	T3	Sigma	30 ng/ml
	Antibiotic-Antimycotic	Invitrogen	1X

### Isolation of OPCs

OPCs were purified from mouse brains using the common shaking culture method for primary OPCs^24,25^. Cortices from E18 and P7 mice were homogenized and after one week of culture, the mixture of glial cells became confluent. Next, the shaking protocol was performed as described previously^26^. Finally, purified OPCs were seeded in 6-well tissue culture plates with a seeding density of 2 × 10^4^. Flasks were incubated at 37 °C and 5% CO_2_ and the OPC culture medium was changed every two days until the cells were ready to perform experiments.

### Immunocytochemistry for OPCs

Purified OPCs were cultured and grown to 80% confluence in 8-well clear tissue culture Millicell EZ SLIDES (Merck Millipore, PEZGS0816). Cells were fixed in 2% PFA for 10 minutes at room temperature, then the cells were incubated for 2 hours in blocking buffer (1% BSA, 4% normal donkey serum and 0.1% triton X-100 in PBS). Primary antibodies were diluted in blocking solution: mouse anti-Olig2 (1:150; Merck Millipore, MAPN50), rabbit anti-NG2 (1:200; Merck Millipore, AB5320), mouse anti-PDGFRα (1:200; Merck Millipore, 05–1135), mouse anti-sox2 (1:100; MAB4423A4; Merck Millipore), mouse anti-nestin (1:200; Merck Millipore, MAB353), mouse anti-beta III tubulin (1:100; Merck Millipore, MAB1637), rabbit anti-MBP (1:300; Merck Millipore, AB980), mouse anti-GFAP (1:200; Merck Millipore, MAB360), rabbit anti-TTR (1:200; ABBIOTC, 250892;) and incubated overnight at 4 °C. After washing, secondary antibodies anti-rabbit Alexa Fluor-488 (Invitrogen, A-11008) or anti mouse Alexa Fluor-555 (Invitrogen, A-21427) were diluted in PBS and incubated for 1.5 hours at room temperature. Sections were then washed 3 times in PBS before being counterstained with 4′,6′-diamidinio-2- phenylindole (DAPI) and mounted with ProLong Gold Antifade mountant reagent (Invitrogen, P36930). Samples were examined using confocal microscopy under a x40 objective lens (E800; Nikon, Champigny-sur-Marne, France, PCM2000).

### Cell proliferation assay (Ki67) for OPCs

Purified OPCs were seeded in 8-well clear tissue culture Millicell EZ SLIDES coated with Geltrex, with a cell density of 5 × 10^4^ per well. After 72 hours, immunohistochemistry was performed using the following markers: the proliferation marker Ki67 was detected using the anti-mouse Alexa-Fluor488 conjugated antibody and the oligodendrocyte marker (Olig2) was detected using the anti-mouse Alexa-Fluor555 conjugated antibody. Next, the samples were analyzed under a confocal microscope. At least ten representative high-power fields (HPF x40 microscope objective) of each well were photographed. The proportion of cells positive for both markers (Ki67 and Olig2) and the cells positive for Olig2 alone were quantified for each field. The mean of ten HPFs was calculated and expressed as the percentage of OPCs undergoing proliferation.

### Cell cycle analysis of OPCs

The BrdU assay^27^ was performed to investigate cell proliferation and to quantify the proportion of cells in each phase of the cell cycle. The purified OPCs were seeded in 6-well clear tissue culture plates coated with Geltrex, with a cell density of 1 × 10^5^ per well. Next, the cells were pulsed with 1 mM BrdU in PBS for one hour. Cells were harvested and the BrdU Flow Kit (BD Pharmingen, 557891) protocol was followed to analyze the cell cycle, according to the manufacturer’s instructions. Finally, the stained cells were acquired on a FACS Canto II flow cytometer (BD Biosciences) and analysed with FlowJo software.

### Chemotaxis migration assay

The chemotaxis migration assay for OPCs was performed using a Boyden chamber^28^. Migration chambers (polyethylene terephthalate 8.0 µm membrane; Merck Millipore, PIEP12R48) were coated with 0.01 mg/ml poly-L-ornithine solution for 1 hour at 37 °C. Next, the coating material was removed and the chambers were rinsed twice with sterile water. About 55 × 10^4^ purified OPCs were seeded in each chamber with 200 μl of OPC Complete medium, and then the migration chambers were placed in 24-well dishes. Medium (750 μl) was added to the bottom well with a chemo-attractant (CXCL12, 200 nM). After 16 hours of incubation at 37 °C in a 5% CO_2_-humidified incubator, the medium was removed and the migration chambers were then fixed with 4% paraformaldehyde (PFA) for 10 minutes at room temperature. Next, the chambers were washed twice with PBS and stained with 0.1% cresyl violet for 30 minutes at room temperature in darkness. Cresyl violet stain was removed and the chambers were washed twice with PBS. Cells that did not migrate were scraped from the upper compartment using a cotton swab. The membrane was photographed for 10 microscopic fields per well at 400x magnification and TIFF images were acquired with a CDD video camera (Canon) mounted on an inverted photomicroscope (Lecia, Wetzlar, Germany). The migrated cells were quantified for each field. The mean of ten HPFs was calculated and expressed as the number of migrated cells.

### Terminal deoxynucleotidyl transferase dUTP nick end labeling (TUNEL) assay for OPCs

Purified OPCs were seeded in 8-well clear tissue culture Millicell EZ SLIDES coated with Geltrex (ThermoFisher, 12760013), with a cell density of 5 × 10^4^ per well. After 72 hours, the medium was removed and the cells were fixed with 4% PFA for 10 minutes and washed three times with PBS. Next, the cells were permeabilized by adding 0.2% Triton X-100 to PBS for 5 minutes. Cell death was detected with a fluorescent cell death detection kit (DeadEnd Fluorometric TUNEL System, Promega. G3250) according to the manufacturer’s instructions. Thereafter, cells were stained with the Olig2 marker by immunohistochemistry. The stained cells were analyzed under a confocal microscope (using x40 objective lens). At least ten representative high-power fields (HPF x40 magnification) of each well were photographed. The proportion of cells positive for both TUNEL-positive nuclei and Olig2 and the proportion of cells positive for Olig2 alone were quantified for each field. The mean of ten HPFs was calculated and expressed as the percentage of apoptotic oligodendroglial cells.

### Tissue preparation for histological analysis

P7, P14, P21 and 12-week-old mice were deeply anesthetized by sodium pentobarbital, weighed and perfused transcardially with 10 mL 0.1 M PBS followed by 10 mL 4% paraformaldehyde (PFA). Next, brains were harvested, weighed and fixed overnight in 4% PFA. The next day, brains were processed, embedded in paraffin blocks and cut by microtome into coronal section with 10 μm thickness.

### Immunohistochemistry

Formalin fixed paraffin embedded (FFPE) sections were dewaxed and rehydrated according to the following protocol: incubated at 60 °C for 60 minutes, immersed in xylene for 3 minutes, then 20 times in fresh xylene, 10 times in absolute alcohol, a further 10 times in fresh absolute alcohol, then immersed 10 times in 70% alcohol, a further 10 times in fresh 70% alcohol and briefly washed in running tap water. Samples were immersed in 10 mM citrate buffer pH 6 and heated at 97 °C for 10 minutes (Dako, PT10027). Then, samples were washed twice in PBS for 5 minutes and a border was marked around the sample using a PAP pen. Samples were covered with 4% paraformaldehyde for 20 minutes at room temperature and washed twice with PBS for 5 minutes. Samples were covered with a blocking buffer containing 10% normal goat serum (ThermoFisher, 16210064) or normal donkey serum (Sigma, D9663) and 0.1% Triton X-100 diluted in PBS, for 2 hours at room temperature. Then, samples were washed twice in PBS for 5 minutes. A primary antibody diluted in blocking buffer was applied to the samples and incubated overnight at 4 °C in a humidified chamber. The primary antibody was removed by washing twice in PBS for 5 minutes each. A secondary antibody was diluted in blocking buffer and incubated at room temperature, away from light, for 2 hours. After washing twice for 5 minutes in PBS, 300 nM 4′,6-diamidino-2-phenylindole dilactate (DAPI) (ThermoFisher, D3571) was applied and incubated at room temperature for 15 minutes. Then, samples were washed twice in PBS for 5 minutes each and mounted with a glass coverslip and fluorescence mounting medium (Dako, S3023).

### Imaging and quantification for formalin fixed sections

Images were scanned using an Olympus VS-120 slide scanning microscope (Olympus, Japan) and images were captured at 20x magnification using an Hamamatsu ORCA-Flash4.0 digital camera (Iwata, Japan). Cells were counted using Cellsens dimension software version 2.2 (Olympus, Japan).

### Antibodies

Primary antibodies: Rabbit anti-TTR IgG (ThermoFisher, PA580196), 1:200; Rabbit anti-Olig2 IgG (Millipore, AB9610), 1:200; Goat anti-Sox10 IgG (R&D Systems, AF2864), 1:500; Mouse anti-APC/CC1 IgG (Millipore, OP80), 1:50; Rabbit anti-AKT IgG (Cell Signaling Technologies, 4691), 1:1000; Rabbit anti-ERK1/2 IgG (Cell Signaling Technologies, 4695), 1:1000; Rabbit anti-pAKT IgG (Ser473) (Cell Signaling Technologies, 9271), 1:2000; Rabbit anti-pERK1/2 IgG (Thr202/Tyr204) (Cell Signaling Technologies, 4370), 1:2000.

Secondary antibodies: HRP-linked anti-rabbit IgG (Cell Signaling Technologies, 7074), 1:2000–20000; Goat anti-mouse Alexa 488 IgG (ThermoFisher, A10667), 1:500; Goat anti-rabbit Alexa 647 IgG (ThermoFisher (A27040), 1:500; Donkey anti-mouse Alexa 488 IgG (ThermoFisher, A21202), 1:500; Donkey anti-goat Alexa 555 IgG (ThermoFisher, A21432), 1:500; Donkey anti-rabbit Alexa 647 IgG (ThermoFisher, A31573), 1:500.

### Tissue preparation for electron microscopy analysis

P7, P14, P21 and 12-week-old mice were deeply anesthetized with sodium pentobarbital. Once anaesthetized, mice were weighed and perfused transcardially with 2% PFA, 2% glutaraldehyde in PBS. Brains were collected and post-fixed in 2% PFA, 2% glutaraldehyde in PBS. Corpus callosum was isolated from 1 mm coronal slices of brain corresponding to the area between 0.0 and 1.3 of bregma. Briefly, after fixation the tissue was washed with 0.1 M cacodylate buffer pH 7.4. Next, samples were immersed in a 1:1 solution of 2% aqueous OsO_4_ and 0.2 M cacodylate buffer pH 7.4. The tissues were dehydrated in ascending grades of ethanol alcohol solution (50%, 70%, 95% then 100% x 2) for 15 minutes each. The slides were then decanted into propylene oxide twice, for 15 minutes each. Samples were then immersed in resin (Epoxy, LR White and Lowicryl) overnight. The next day, the samples were immersed in fresh resin for 2 hours. The ‘blocks’ were incubated for 48 hours in an oven at 60 °C. Finally, the ultra-thin sections were placed into a transmission electron microscope (TEM) grid and stained with 1% uranyl acetate and lead citrate. All the ultra-thin sections were examined using a JEOL JEM 1010 (Jeol Ltd., Tokyo, Japan) TEM. Three serial sections at distances of >50 μm apart, from 3 coded mouse brain sections, were analyzed per genotype from each age group. A minimum of 100 myelinated axons were counted per animal. The *g* ratio of axons was calculated by measuring the ratio of the inner diameter of the axon alone to the outer diameter of the fiber (axon and myelin).

### RT-PCR analysis

mRNA was isolated with an RNeasy Kit (Qiagen, 74104) and subjected to RT-PCR with the Superscript II kit (Invitrogen). The total volume of the PCR reaction was 25.0 µl in each tube, which contained 12.5 μl Qiagen HotStartTaq Mastermix, 2.0 µl 4.0 μM primers, the appropriate volume of cDNA, and ultra-pure water to bring the final volume to 25.0 µl. Samples were loaded into a 2720 Thermal Cycler (Applied Biosystems) and specific PCR conditions were used based on the primer composition. The PCR product was mixed with 2.0 µl 6x loading buffer (Bioline, Bio-37068) and 0.5 µl Gel Red (Biotium, 41003), then loaded into the well for agarose gel electrophoresis. The gel was photographed under UV light using the FluorChem SP Digital Imaging System (Alpha Innotech Corporation, San Leandro, California, USA). Fluorescence density of each PCR-amplified band was normalized with the corresponding value of the GAPDH cDNA band.

### Optic nerve dissection

Forceps were used to detached eyes from euthenased mice. Then, fine scissors were used to dissect optic nerves from eyes, avoiding muscle and connective tissue. Optic nerves were transferred to collection tubes and snap frozen in liquid nitrogen. Then, samples were transferred to a − 80 °C freezer for downstream applications.

### Protein extraction

Working on a bed of dry ice, optic nerves from mice (14–20 weeks of age) of the same genotype were pooled and crushed in a liquid nitrogen cooled mortar and pestle. Powdered tissue was suspended in radioimmunoprecipitation assay (RIPA) (Cell Signaling Technologies, 9806S) buffer containing 1:100 protease/phosphatase inhibitors (Cell Signaling technologies, 5872S). The suspension was mixed and homogenised through a 26-gauge needle attached to a 1 ml syringe. The homogenate was centrifuged at 1000 × g at 4 °C for 20 minutes. The supernatant lysate was collected and stored at −80 °C for later use.

### Measurement of protein concentration

A Pierce bicinchoninic acid assay (BCA) protein assay kit (Thermo Scientific, 23225) was used to determine the total concentration of protein in a sample lysate by measuring absorbance at 562 nm on the microplate reader (BMG Labtech, Clariostar) and comparing it to the absorption of the known protein standard: bovine serum albumin (BSA).

### Sodium dodecyl sulfate polyacrylamide gel electrophoresis (SDS-PAGE)

A volume containing 5 µg of protein, 1 x sample buffer (ThermoFisher, NP0007), 1 x reducing agent (ThermoFisher, NP0004) and ddH2O were denatured by heating at 95 °C for 5 minutes (or 100 °C for 15 minutes for TTR monomer detection). The sample mixture was cooled on ice for 5 minutes before being briefly centrifuged. Samples and protein ladder (ThermoFisher, 26634) were loaded into 4–12% Bis-Tris pre-cast protein gels (ThermoFisher, NP0335) or 12% Bis-Tris pre-cast protein gels for TTR controls (ThermoFisher, NP0341), in MOPS running buffer supplemented with 1:400 antioxidant (ThermoFisher, NP0005). Electrophoresis (Biorad, Mini-protean) was set to 150 V for 1.5 hours at room temperature.

### Western blots

Following separation by SDS-PAGE, proteins were transferred onto polyvinylidene fluoride (PVDF) membranes using transfer buffer (20% methanol, 70% MilliQ water, 0.3% Tris, 0.2 M glycine). The membrane was blocked in 5% skim milk in tris-buffered saline with 0.1% Tween (TBST) for 30 minutes at room temperature. Primary antibodies were diluted in 5% skim milk with TBST and incubated with the membrane overnight on a rocker at 4 °C, then washed four times in TBST for 10 minutes at room temperature. Secondary HRP conjugated antibodies diluted in 5% skim milk in TBST were added for 2 hours at room temperature. The membrane was washed 4 times in TBST for 10 minutes per wash. An enhanced chemiluminescence (ECL) reagent (Cell Signaling Technologies, 6883 S) was applied to the membrane for 1 minute at room temperature and then imaged under ultraviolet light (Biorad, Chemidoc XRS).

### Immunoprecipitation

An antibody-antigen complex was prepared by adding 100 µg of protein lysate and primary antibody in RIPA buffer to a total volume of 1 ml, then incubated overnight at 4 °C on a rotary tube mixer. The following day, 50 µl of protein-G magnetic beads (Millipore, LSKMAGG10) were dispensed into a 1.5 ml collection tube and placed on a magnetic rack (Millipore, 20–400). The storage substrate was removed and the beads were washed twice in 500 µl PBS with 0.1% Tween-20 (PBST). The mixture was briefly vortexed and centrifuged between washes and the supernatant was discarded. Next, the antibody-antigen complex was mixed with the magnetic beads and incubated overnight on a rotary tube mixer at 4 °C. The next day, the mixture was briefly centrifuged and washed 3 times in PBST. The elution was resuspended in 25 µl RIPA buffer with reducing agent (ThermoFisher, NP0004) and sample buffer (ThermoFisher, NP0007). Next, the samples were denatured by heating at 95 °C for 10 and loaded into 4–12% Bis-Tris pre-cast protein gels for SDS-PAGE.

### Western blotting: cell culture lysates

Cell lysates were prepared in a buffer containing 150 mM sodium chloride, 1.0% Triton X-100 and 50 mM Tris pH 8.0 and protein quantitation was performed^29^. Proteins from cell lysates (5 µg of total protein) were separated by electrophoresis in SDS-polyacrylamide gels and blotted to PVDF membranes using the iBLOT system (Invitrogen, IB4010-01). Membranes were blocked with 5% skim milk in PBS-T then incubated with primary antibody (rabbit anti-TTR, 1:200; ABBIOTC, 250892), washed twice with TBS-T and then incubated with a peroxidase-linked secondary antibody (1:1000). The membrane was washed with TBS-T x 3 then with TBS before being developed using an ECL detection system (GE Healthcare, 5995578). The membrane was exposed to X-ray film for 10 minutes and developed in the dark room.

### Concentration of proteins from cell culture

Proteins in the cell medium were concentrated 20-fold using a 10K Centriprep centrifugal filter from Millipore at 4000 × *g* for 15 minutes at 4 °C. The concentrated media were analyzed for the presence of TTR secreted by OPCs by western blotting.

### Flow cytometry

Cells (1 × 10^6^) were washed twice with HEPES-buffered saline, treated with Accutase (ThermoFisher, A1110501), and fixed for 20 minutes at room temperature in 2.0% PFA. Cell membranes were permeabilized by adding 0.1% TritonX-100 in PBS for 20 minutes at room temperature. Cell pellets were collected and incubated with primary antibodies diluted in blocking solution: mouse anti-Olig2 (1:200; Merck Millipore, MAPN50), mouse anti-beta III tubulin (1:150; Merck Millipore, MAB1637), mouse anti-GFAP (1:200; Merck Millipore, MAB360), rabbit anti-TTR (1:300;; ABBIOTC, 250892) and incubated overnight at 4 °C. After washing, secondary antibodies anti-rabbit Alexa Fluor-488 (Invitrogen, A-11008) or anti-mouse Alexa Fluor-555 (Invitrogen, A-21427;), were diluted in PBS and incubated for 1.5 hours at room temperature. Flow cytometric evaluation was conducted within 5 minutes. Cells were analyzed by flow cytometry using an EPICS XL-MCL FACScan (Becton–Dickinson, Mountain View, CA, United States).

### Immunohistochemistry for cryosections

Cryosections (sectioned at 12 µm) from 4% paraformaldehyde (PFA) fixed samples were dried for 30 minutes at room temperature and permeabilized in a solution of PBS-0.1% Triton-X (PBS-T) for 10 minutes. Samples were immersed in a blocking buffer made from 10% normal donkey serum (NDS) (Sigma, D9663)/1% bovine serum albumin (BSA) (Thermo Fisher, B14)/PBS for 1 hour at room temperature. Samples were incubated with primary antibody diluted in blocking buffer overnight at 4 °C with gentle agitation. The primary antibody was removed, and samples were washed (x3) in PBS for 10 minutes each. In the next steps, samples were protected from light whenever possible. A secondary antibody was diluted in 1% NDS/1% BSA and incubated 2 hours. Samples were washed in PBS (3 × 10 minutes). Samples were incubated in 300 nM DAPI for 7 minutes. Samples were washed in PBS for 10 minutes, repeated three times and mounted on glass slides using ProLong-Gold antifade mounting media (Thermo Fisher, P10144). Mounted samples were imaged using a Leica TCS-SP5 confocal microscope (Nussloch, Germany). Four images were taken of each brain sample (from n = 5–6 mice per genotype). Antibodies and dilutions: mouse anti-PH3 IgG (Millipore 05598) 1:400; rabbit anti-Olig2 IgG (Millipore AB9610) 1:200; donkey anti-mouse Alexa 488 IgG (ThermoFisher A21202) 1:500; donkey anti-rabbit Alexa 594 IgG (ThermoFisher A21207) 1:500.

### Statistical analyses

Statistical analyses were performed using version 9 of SPSS and GraphPad Prism version 6. A one-way ANOVA was performed with post hoc analysis using Sidak’s test for multiple comparisons of counted cell numbers *in situ* over the various age groups, or a Student’s *t*-test for all comparisons in culture between TTR null and wild type mouse groups. All data are presented as mean ± standard error of mean (SEM). A *P* value of <0.05 was considered statistically significant.

## Results

### TTR null mice have enlarged brains during postnatal development that correlates with a hypermyelinating phenotype

**Figure 1:**
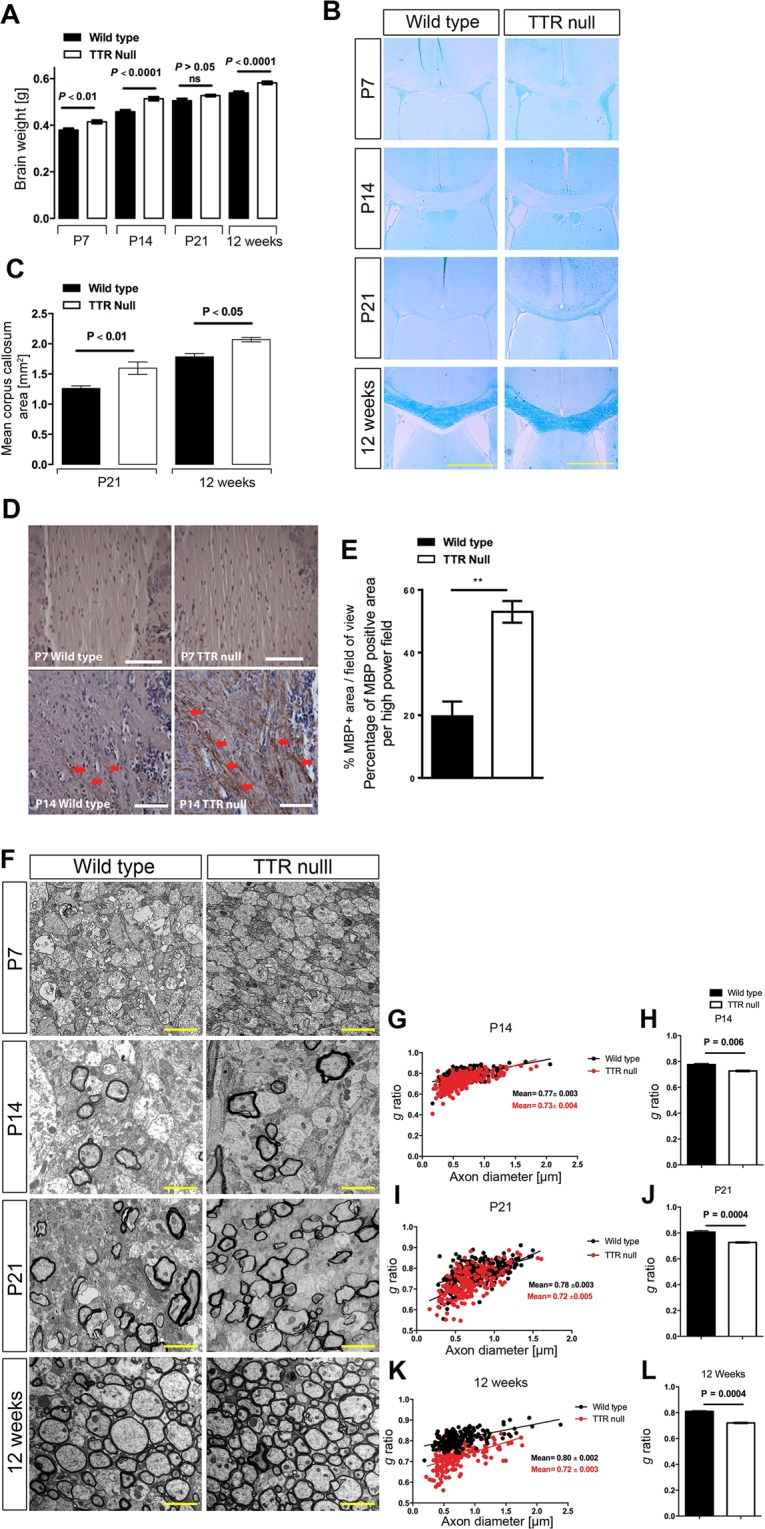
TTR null mice have a hypermyelination phenotype. (**A**) Comparison of the size of the myelinated area of the corpus callosum between wild type and TTR null mice. Coronal brain sections of 10 µm thickness corresponding to the area between 0.6 and 1.3 bregma were analysed for mice aged P7, P14, P21 and 12 weeks for myelin staining using LFB. Myelin was not detected in the corpus callosum of P7 or P14 mice. However, at P21 the entire corpus callosum was clearly stained (myelinated) in TTR null mice, but not in wild type mice. For 12-week-old mice, LFB stained the entire corpus callosum in both wild type and TTR null mice (scale bar 2 mm). (**B**) Comparison of brain weights between TTR null and wild type mice. The brain weights of TTR null mice at ages P7, P14, and 12 weeks were heavier than age-matched wild type mice (but not significantly different for P21 mice). All data were expressed as the mean ± SEM. Statistical comparisons were performed using a one-way ANOVA with post hoc analysis using Sidak’s test as appropriate (n = 6 mice per group were assessed and *P* < 0.05 was considered statistically significant). (**C**) Graphical summary of the comparison of the size of the myelinated area of the corpus callosum between P21 and 12-week-old wild type and TTR null mice using LFB. All data were expressed as the mean ± SEM. Statistical comparisons were performed using a one-way ANOVA with post hoc analysis using Sidak’s test as appropriate (n = 5 mice per group were assessed and *P* < 0.05 was considered statistically significant). (**D**) Detection of myelin within the corpus callosum at P7 and P14 for wild type and TTR null mice by MBP immunostaining. Coronal brain sections of 10 µm thickness, corresponding to the area between 0.6 and 1.3 bregma for wild type and TTR null mouse corpus callosum were stained with a polyclonal anti-MBP antibody. MBP was not detected in P7 mice. P14 mice revealed MBP-immunoreactive myelinated fibres (red arrows) in the corpus callosum indicating that myelination had begun in earnest in the TTR null mice (scale bar 200 µm). (**E**) Quantification of the MBP positive area per 0.3 mm^2^ for P14 mice. All data were expressed as the mean ± SEM. Statistical comparisons were performed using a Student’s *t*-test (n = 3 mice were analyzed at this time point and *P* < 0.05 was considered statistically significant). (**F**) Representative EM images of the corpus callosum from P7, P14, P21 and 12 weeks-of-age respectively, for both wild type and TTR null mice. As expected, myelin cannot be detected in either genotype at P7. However, from P14 to 12 weeks-of-age, myelin thickness is visibly increased in TTR null compared to wild type mice. (**G**) Measurement of myelin thickness and calculation of *g* ratios in P14 wild type and TTR null mice (n = 3 mice per genotype, G- ratios were calculated from 100 axons per mouse. Graphical illustration of the relationship between *g* ratio of individual axons and axon diameter, with regression lines for P14 wild type and TTR null mice. (**H**) Histogram of *g* ratio comparison between P14 TTR null and wild type mice. All data were expressed as the mean ± SEM. Statistical comparisons were performed using a Student’s *t*-test (*P* < 0.05 was considered s*t*atistically significant). (**I**) Measurement of myelin thickness and calculation of *g* ratios in P21 wild type and TTR null mice (n = 3 mice per genotype, *g* ratios were calculated from 100 axons per mouse). Graphical illustration of the relationship between *g* ratio of individual axons and axon diameter, with regression lines for P21 wild type and TTR null mice. (**J**) Summary histogram of the *g* ratio comparison between P21 TTR null and wild type mice. All data were expressed as the mean ± SEM. Statistical comparisons were performed using a Student’s *t*-test (*P* < 0.05 was considered statistically significant). (**K**) Measurement of myelin thickness and calculation of *g* ratios in 12-week-old wild type and TTR null mice. n = 3 mice per genotype. *g* ratios were calculated from 100 axons per mouse. Graphical illustration of the relationship between *g* ratios of individual axons and axon diameter, with re*g*ression lines for 12-week-old wild type and TTR null mice. (**L**) Summary histogram of the *g* ratio comparison between 12-week-old wild type and TTR null mice. All data were expressed as the mean ± SEM. Statistical comparisons were performed using a Student’s *t*-test (*P* < 0.05 was considered statistically significan*t*)^30^.

Wild type and TTR null mice at P7, P14, P21 and 12 weeks of age, were euthanized and their brains weighed after resection of the medulla at the caudal end of the pons and the olfactory bulbs. The brains of P7 TTR null mice (0.4145 ± 0.0067 g, n = 6) were heavier than those of P7 wild type mice (0.3793 ± 0.0029, n = 6). Additionally, there was an approximately 12% increase in the brain weight of P14 TTR null mice (0.5138 ± 0.0074 g, n = 6) compared to age-matched wild type mice (0.4582 ± 0.0072 g, n = 6). However, there was no significant difference in the brain weight of P21 TTR null mice compared to wild type mice of the same age. Finally, the brains of 12-week-old TTR null mice (0.5822 ± 0.006 g, respectively, n = 6) were significantly heavier than those of wild type mice of the same age (0.5388 ± 0.0064 g, n = 6) (Fig. 1A,^30^).

Myelin in the corpus callosum was histochemically analyzed using Luxol fast blue (LFB) staining. Coronal brain sections (10 µm-thick) corresponding to the area between 0.6 and 1.3 bregma (demonstrating the entire mid-sagittal continuation of the corpus callosum) were selected and stained, with myelin pallor evident in P7 and P14 mice (Fig. 1B). However, LFB weakly stained myelin along the mediolateral extent of the corpus callosum in P21 mice (Fig. 1B). At this age, the corpus callosum of TTR null mice had a significantly larger myelinated area compared to that of wild type mice (Fig. 1C). Finally, at the 12-week-old developmental time-point, LFB staining of myelin was intensely distributed along the entire corpus callosum of both TTR null and wild type mice (Fig. 1B). However, the LFB-stained myelinated area was greater in the brains of TTR null mice compared to brains of wild type mice (Fig. 1C).

To detect ontogenic differences in myelination of the corpus callosum of TTR null compared with wild type mice, immunohistochemistry was performed for myelin basic protein (MBP) at P7 and P14. MBP was not detected in the corpus callosum of P7 TTR null or wild type mice (Fig. 1D). However, MBP-positive fibers were detected in the medial region of the corpus callosum of both P14 TTR null and wild type mice (Fig. 1D, arrows), with more intensely-stained fibers visible in TTR null compared to wild type mice (Fig. 1D, arrows). This was significantly evident when the size of the anti-MBP immunopositive area of the corpus callosum of P14 TTR null and wild type brain sections were measured (Fig. 1E).

To investigate the extent of myelination at an ultrastructural level, the thickness of the myelinated axons of wild type and TTR null mice during postnatal development was measured by analyzing the *g* ratios of corpus callosum axons following electron microscopy (EM). At P7, EM images showed unmyelinated axons in the corpus callosum for both TTR null and wild type mice, as expected at this developmental stage^31^ (Fig. 1F). *G* ratio analysis for P14 mice showed significant differences among the strains (*P* = 0.006), with TTR null mice having a lower *g* ratio (0.73 ± 0.004) compared with wild type mice (0.77 ± 0.003). The normal range for *g* ratio values in the mouse corpus callosum is 0.75–0.81^32,33^, indicating that hypermyelination occurred in P14 TTR null mice (Fig. 1G,H).

At P21, the *g* ratio analyses illustrated significant differences between the genotypes (*P* = 0.0004), showing a significant reduction in TTR null mice (0.72 ± 0.005) compared to wild type mice (0.78 ± 0.003). The values obtained indicated that hypermyelination occurred at P21 in TTR null mice (Fig. 1I,J).

At 12 weeks of age, the *g* ratio analyses identified significant differences between the genotypes (*P* = 0.0004), with a lower *g* ratio again evident in the TTR null mice (0.72 ± 0.003) when compared to wild type mice (0.80 ± 0.002). The values obtained indicated that hypermyelination was prominent at 12 weeks of age in TTR null mice (Fig. 1K,L).

### TTR null mice have increased numbers of mature CC1-positive in the corpus callosum during early stages of postnatal development

**Figure 2:**
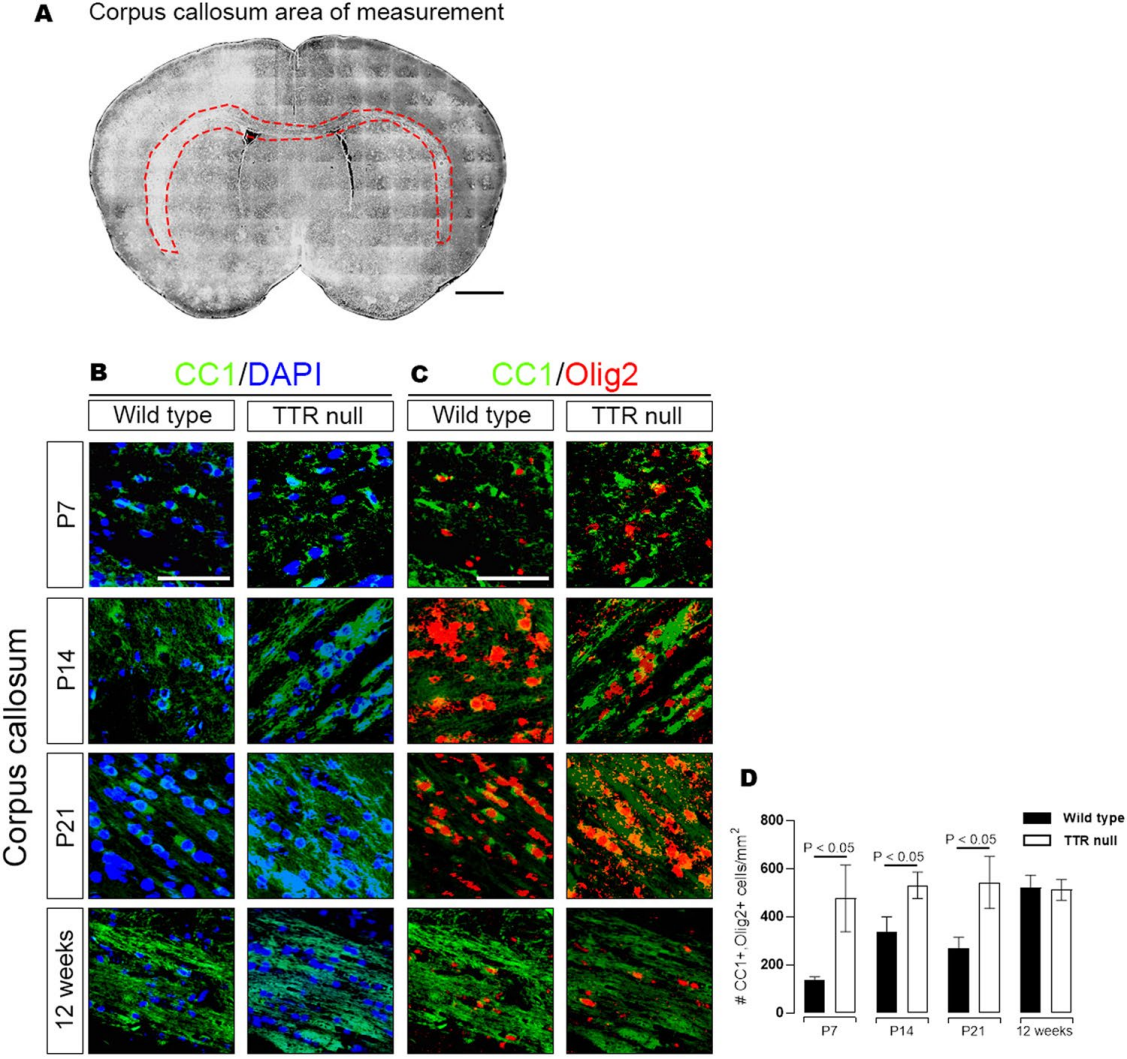
TTR null mice have increased numbers of mature CC1-positive oligodendrocytes in the corpus callosum during early stages of postnatal development. (**A**) Measured area outlining the corpus callosum used in cell density analyses of Olig2, CC1 and DAPI positive cells from wild type and TTR null mice (scale bar 1 mm). (**B**) Images of coronal brain sections (10 µm) between 0.6 to 1.3 bregma representing cells stained for CC1 and DAPI in wild type and TTR null mice from ages P7, P14, P21 and 12 weeks (scale bar 200 µm). **(C)** Images of coronal brain sections (10 µm) between 0.6 to 1.3 bregma representing cells stained for CC1 and Olig2 in wild type and TTR null mice from ages P7, P14, P21 and 12 weeks (scale bar 200 µm). **(D)** A semi-quantitative analysis of CC1/Olig2 positive cells counted per mm^2^ throughout the corpus callosum of wild type and TTR null mice at ages P7, P14, P21 and 12 weeks. Data were expressed as the mean ± SEM. Age groups of mouse genotypes (n = 6 per group) were compared using an unpaired Student’s *t*-test (*P* < 0.05 was considered statistically significant).

To determine whether late oligodendroglial lineage cells contributed to the hypermyelination phenotype observed in TTR null mice, numbers of CC1-positive (a marker for mature oligodendrocytes) cells were quantified in the corpus callosum of wild type and TTR null mice (Fig. 2B). Coronal brain sections with 10 µm thicknesses corresponding to the area of genu corpus callosum between 0.6 and 1.3 bregma were analyzed. Numbers of cells that were CC1-positive and Olig2-positive (a marker for all oligodendrocytes) were counted from a traced cross section (Fig. 2A) of the entire corpus callosum in wild type and TTR null mice at ages P7, P14, P21 and 12 weeks (n = 6 per genotype/age group). Representative images for sections co-stained for DAPI and CC1 (Fig. 2B) and for CC1 and Olig2 (Fig. 2C) are shown.

At P7, TTR null mice exhibited a significantly higher (*P* < 0.05) density (~ 477 cells/mm^2^) of CC1-positive cells in the corpus callosum compared to wild type mice (~ 140 cells/mm^2^) (Fig. 2C). At P14, TTR null mice similarly displayed a significantly higher (*P* < 0.05) density (~ 531 cells/mm^2^) of CC1-positive cells in the corpus callosum compared to wild type mice (~ 340 cells/mm^2^) (Fig. 2C). Similarly, at age P21, TTR null mice displayed a significantly (*P* < 0.05) higher density of CC1-positive cells (~ 543 cells/mm^2^) compared to wild type mice (269 cells/mm^2^) (Fig. 2C). Whereas, 12-week-old wild type and TTR null mice did not display a significant difference (*P* > 0.05) in CC1-positive cell densities in the corpus callosum (~ 522, 513 cells/mm^2^ respectively) (Fig. 2C). The greatest observed difference in CC1-positive densities between genotypes from the four age groups were the P7 TTR null mice which represented an ~ 3.4-fold higher CC1-positive cell density than the wild type mice (Fig. 2C).

### Increase in the proliferation rate of NSCs in cultured neurosphere colonies isolated from the SVZ of P21 TTR null mice and their preferential differentiation toward a glial lineage

**Figure 3:**
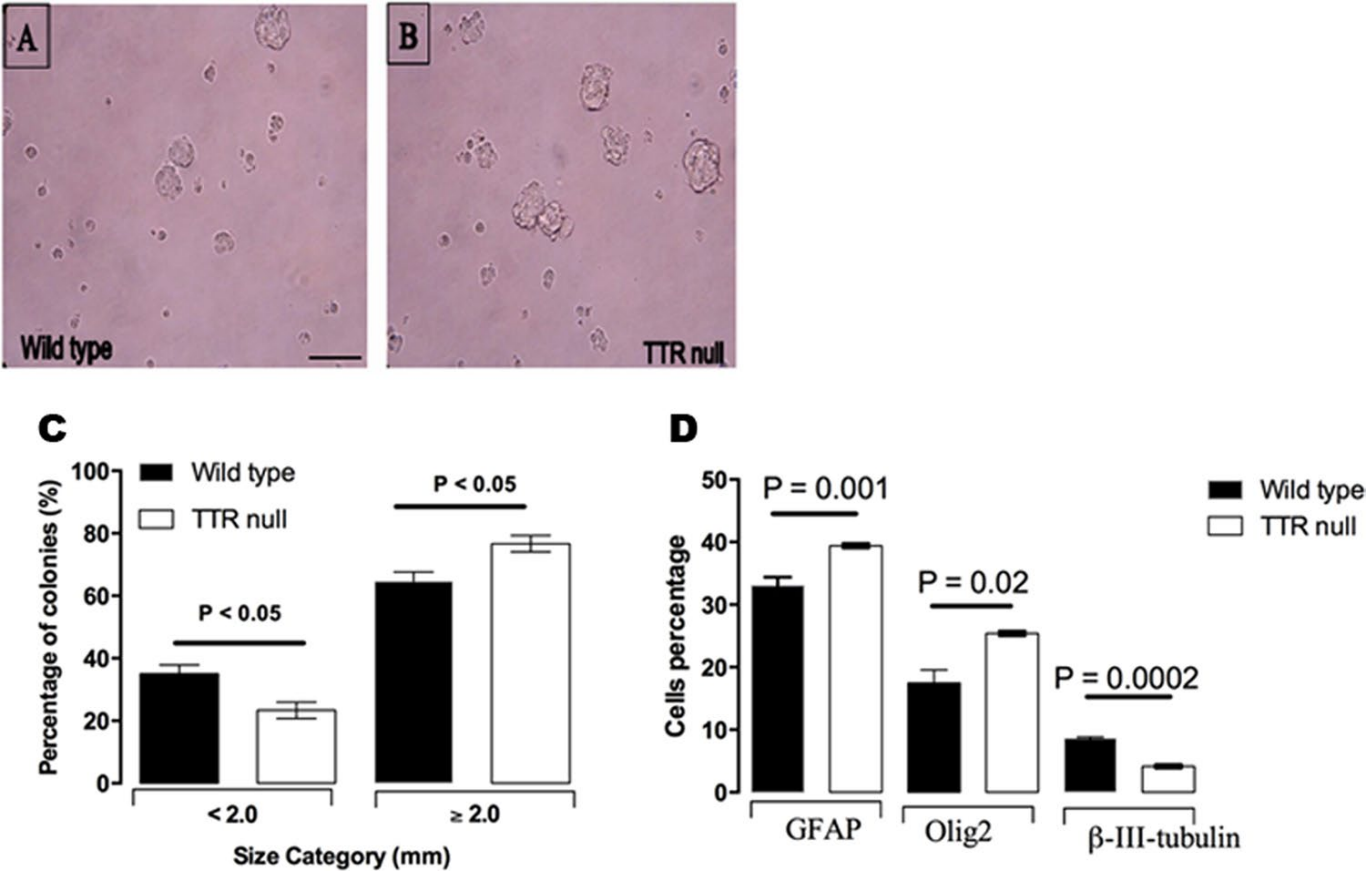
TTR influences OPC differentiation. Representative images of neurospheres in 3D culture, isolated from SVZ derived NSCs of P21 (**A**) wild type and (**B**) TTR null mice. Scale bar 5 mm. (**C**) Quantitation of the potency of the NSC colonies isolated from the SVZ of P21 mice by neural colony-forming cell assays. There was a significant increase in the number of colonies with an average diameter ≥ 2 mm that were isolated from the SVZ of P21 TTR null mice when compared with wild type at the same age. Increases corresponded with a significant decrease in the number of colonies with an average diameter < 2 mm that were isolated from TTR null mouse SVZ compared to wild type. The colonies ≥ 2 mm in diameter are “NSC derived” and have self-renewal and multi-potential capabilities. Colonies < 2 mm diameter are “progenitor derived”. All data were expressed as the mean ± SEM. Statistical comparisons were performed using a one-way ANOVA with post hoc analysis using Sidak’s test as appropriate (data derived from n = 4 independent experiments per genotype and *P* < 0.05 was considered statistically significant). (**D**) Characterization of NSCs isolated from the SVZ of P21 TTR null mice and differentiated into the three neural lineages: oligodendrocytes, astrocytes and neurons. Nuclei were stained with DAPI; oligodendrocytes were stained with anti-Olig2; neurons were stained with anti-β-III-tubulin; astrocytes were stained with anti-GFAP. Lack of TTR promotes NSCs to differentiate into glial precursor cells. Differentiation assay for NSCs isolated from the SVZ of P21 TTR null mice showed a greater proportion of cells differentiating into a glial lineage, whereas the equivalent cells from wild type mice had a greater proportion differentiating into a neuronal lineage. All data were expressed as the mean ± SEM. Statistical comparisons were performed using a one-way ANOVA with post hoc analysis using Sidak’s test as appropriate (n = 3 independent experiments and *P* < 0.05 was considered statistically significant). (Modified from^30^).

The data above demonstrated that there exists an increase in oligodendrocyte numbers in major commissural fibres within the CNS of TTR null mice compared with wild type mice. Therefore, to investigate a potential role for TTR in proliferation of NSCs, we isolated SVZ cells from P21 wild type and TTR null mice and performed neural-colony forming assays. NSCs were plated in uncoated six-well plates to proliferate and make small colonies of cells within one week after plating. After two weeks, colonies of different sizes were able to be distinguished. Thus, after 14 days, colonies were classified into two categories: (1) less than 2 mm in diameter, (2) ≥ 2 mm in diameter. Colonies smaller than 2 mm diameter were considered as progenitor derived and colonies ≥ 2 mm in diameter were considered as NSC derived^34^. The proportion of colonies ≥ 2 mm in diameter to the total number of colonies in each well was calculated. There was a significant increase in the fraction of TTR null NSC colonies with a size ≥ 2 mm in diameter compared to the wild type colonies (Fig. 3A, modified from^30^). These data suggest that the NSCs isolated from the SVZ of P21 TTR null mice have a greater potential for proliferation compared to NSCs isolated from the SVZ of P21 wild type mice.

Since NSCs are an important endogenous source of OPCs and subsequently oligodendrocytes^35,36^, we investigated whether the absence of TTR could affect the differentiation of NSCs into OPCs and oligodendrocytes, or into the other neural lineages. NSCs were harvested from the SVZ of P21 wild type and TTR null mouse brains, then cultured to proliferate and differentiate into each of the three neural lineages. The NSCs isolated from the SVZ of P21 mouse brains were cultured using two different systems, either by monolayer or neurosphere culture systems, for two weeks. When the cells were directed to differentiate into astrocytes, neurons or oligodendrocytes for three weeks, flow cytometric analysis showed that differentiated Olig2-positive oligodendroglial lineage cells were significantly enhanced in NSCs from TTR null mice with ~ 25% of all neural lineages exhibiting Olig2 expression compared to ~ 19% observed from differentiated NSCs from wild type mice (Fig. 3B). Also, there was a significant increase in the number of TTR null NSCs that differentiated toward glial fibrillary acidic protein (GFAP)-positive astrocytes (~ 39%) when compared to NSCs isolated from wild type mice (~33%). Importantly, there was a significant decrease in the number of beta-III tubulin-positive neurons in the TTR null cultured cells (~ 4%) compared to wild type differentiated NSCs (~ 9%) (Fig. 3B). These data indicate that the absence of TTR promotes differentiation of the NSCs into the glial lineage at the expense of differentiation into the neuronal lineage.

### Absence of TTR promotes OPC proliferation

**Figure 4:**
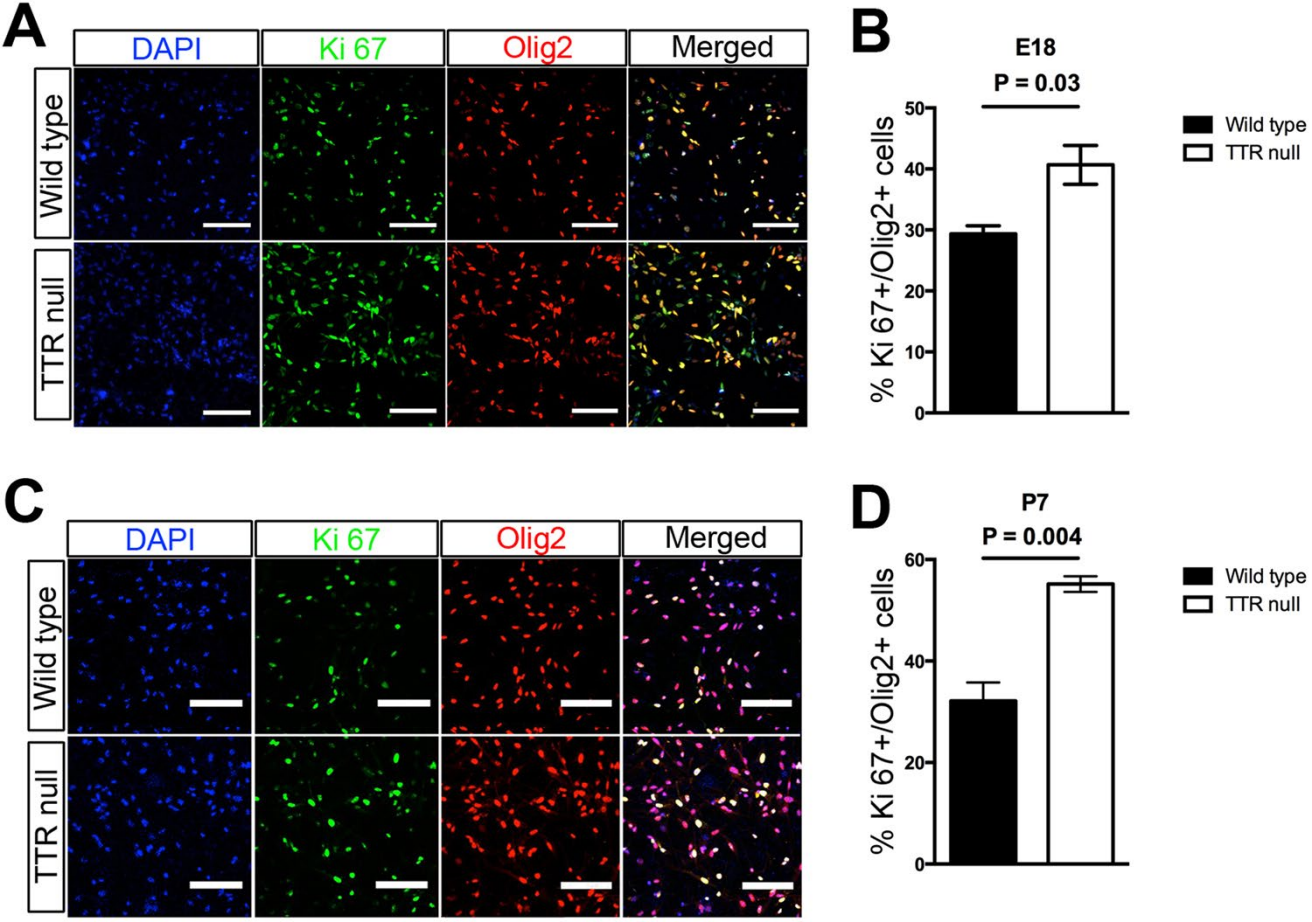
TTR alters the cell cycle of OPCs. (**A**) Absence of TTR enhances proliferation of OPCs isolated from the cerebral cortices of E18 TTR null mice. Upper panel: wild type mice; lower panel: TTR null mice. Nuclei were stained with DAPI; OPCs were stained with polyclonal anti-Ki67 (proliferation marker; green) and monoclonal anti-Olig2 (OPC marker; red) (scale bar 100 µm). (**B**) Semi-quantitative analysis of cells double labeled for Ki67 and Olig2 from cerebral cortices of E18 wild type and TTR null mice. There was a significant increase in the number of proliferating Olig2 positive cells isolated from TTR null mice compared wild type mice. All data were expressed as the mean ± SEM. Statistical comparisons were performed using Student’s *t*-test (n = 4 were mice per genotype and *P* < 0.05 was considered statistically significant). (**C**) Absence of TTR enhances proliferation of OPCs isolated from cortices of P7 TTR null mice. Upper panel: wild type mice; lower panel: TTR null mice. Nuclei were stained with DAPI; polyclonal Ki67 antibody (green); anti-Olig2 monoclonal antibody (red) (scale bar 100 µm). (**D**) Semi-quantitative analysis of cells double labeled for Ki67 and Olig2 from the cerebral cortices of P7 wild type and TTR null mice. There was a significant increase in the number of proliferating Olig2 positive cells isolated from TTR null mice compared to wild type mice. Data were expressed as the mean ± SEM. Statistical comparisons were performed using Student’s *t*-test (n = 4 mice per genotype and *P* < 0.05 was considered statistically significant)^30^.

In order to confirm the effects of the absence of TTR on OPC proliferation, OPCs were isolated from cerebral cortices of E18 and P7 wild type and TTR null mice. The isolated and purified OPCs were cultured and expanded for one week, to limit the chance of any genetic or phenotypic drift that could develop with additional passages. The OPCs were then double labeled for Olig2 and Ki67 to detect proliferating OPCs. The results demonstrated that compared with OPCs isolated from the cortices of the E18 wild type mice, there was a significant increase (~ 23%) in the number of proliferating cells isolated from the cortices of E18 TTR null mice (Fig. 4A,B,^30^). Also, there was a substantial increase (~19%) in the number of proliferating OPCs isolated from the cortices of P7 TTR null compared to the P7 wild type mice (Fig. 4C,D). These data are consistent with the notion that an increase in the number of OPCs in TTR null compared to wild type control mice is contiguous with their endogenous capacity to proliferate in culture.

**Figure 5:**
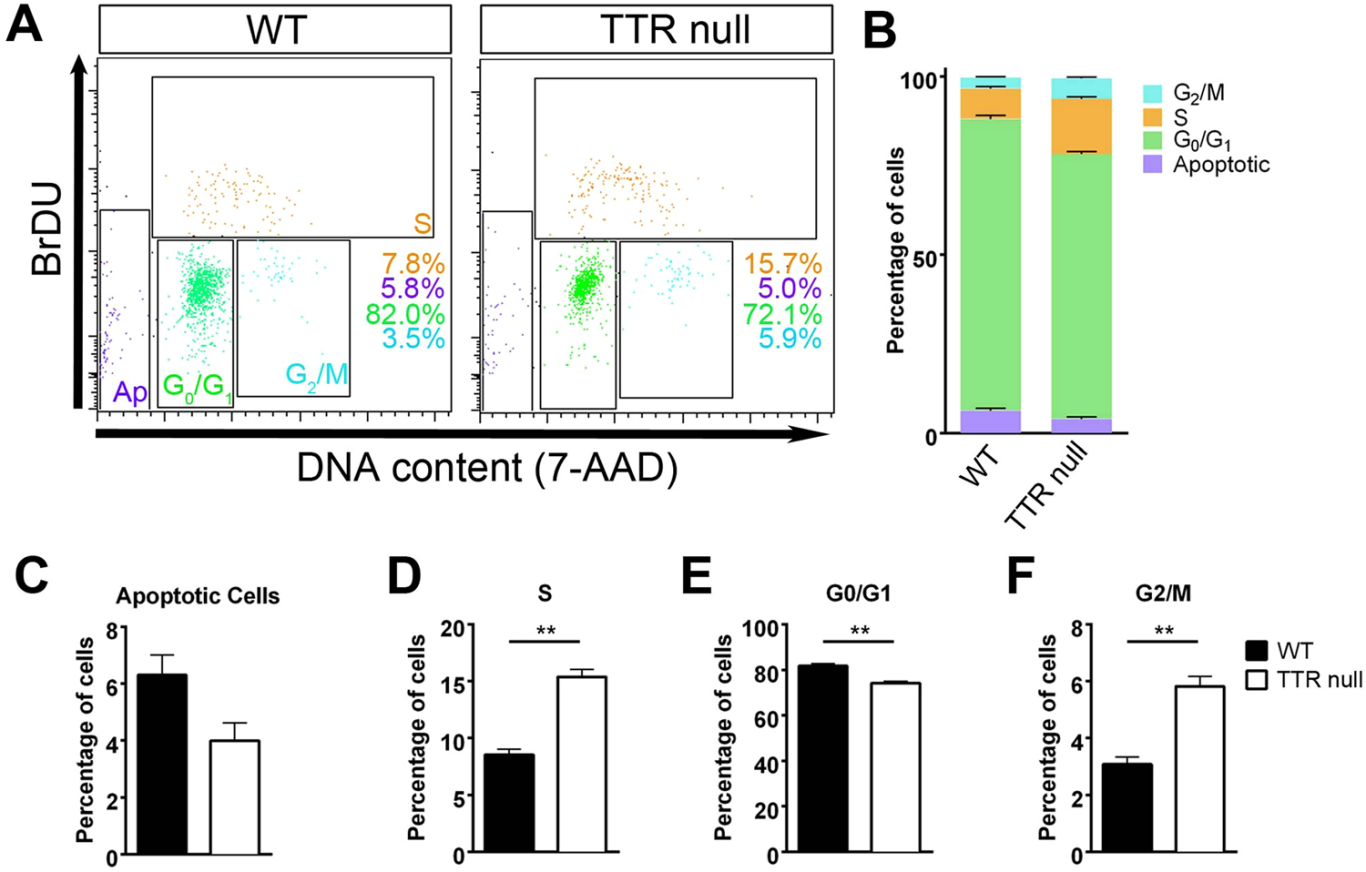
Increased numbers of proliferating OPCs isolated from cortices of P7 TTR null compared to P7 wild type mice. (**A–F**) Flow cytometric analyses of BrdU-mediated cell cycle for OPCs isolated from P7 TTR null and wild type (WT) mice. (**A**) Representative dot plots following BrdU incorporation (Ap: apoptotic cells). **(B)** Percentage of OPCs in each stage of the cell cycle. (**C**) There was no significant difference in the number of apoptotic cells in OPCs from TTR null versus wild type mice (*P* = 0.0711). (**D**) There was a major increase in the population of OPCs in the S phase from TTR null mice (15%) compared to wild type mice (7%) (*P* = 0.0011). (**E**) There was a significant decrease in the population of OPCs cells in G0/G1 phase for OPCs isolated from cortices of TTR null mice (77%) versus wild type mice (84%) (*P* = 0.0031). (**F**) There was a substantial increase in the population of OPCs in G2/M phase in TTR null OPCs (6%) versus 4% in wild type mouse OPCs (*P* = 0.0034). All data were expressed as the mean ± SEM. Statistical comparisons were performed using Student’s *t*-test (n = 3, **P < 0.01, *P* < 0.05 was considered statistically significant)^30^.

To interrogate the specific effect that the absence of TTR exerts on the OPC cycle, we performed bromodeoxyuridine (BrdU) incorporation and flow cytometric analysis using OPCs isolated from the cerebral cortices of P7 mice that had been cultured for one week. We identified a significant increase in the population of TTR null OPCs within S phase (~15%) compared to those isolated from wild type mice (~7%; Fig. 5A,B,D; *P* < 0.01 wild type versus TTR null,^30^). This was accompanied by a significant increase in the OPC population in G2/M phase (~6% in TTR null versus 4% in wild type OPCs; Fig. 5A,B,F), consistent with increased progression from S phase to G2/M phase. Furthermore, there was a significant decrease in the population of TTR null OPCs in G0/G1 phase (~77%) versus those isolated and cultured from wild type mice (~84%; Fig. 5A,B,E) suggesting a decrease in the post-mitotic capacity of TTR null OPCs at this stage of development. Finally, we identified a reduced number of apoptotic cells amongst the cultured TTR null OPCs (~4%) compared with those from wild type mice (~6%), despite there being no statistical significance reached (Fig. 5A,B,C; *P* = 0.0711). Nonetheless, these results imply that the absence of TTR may affect the progression from S phase to G2/M phase, which, in turn, accelerates OPC proliferation and that initially during development the absence of TTR limits the exit of OPCs from the cell cycle toward post-mitotic differentiation.

### Absence of TTR in OPCs potentiates their migration in culture

**Figure 6:**
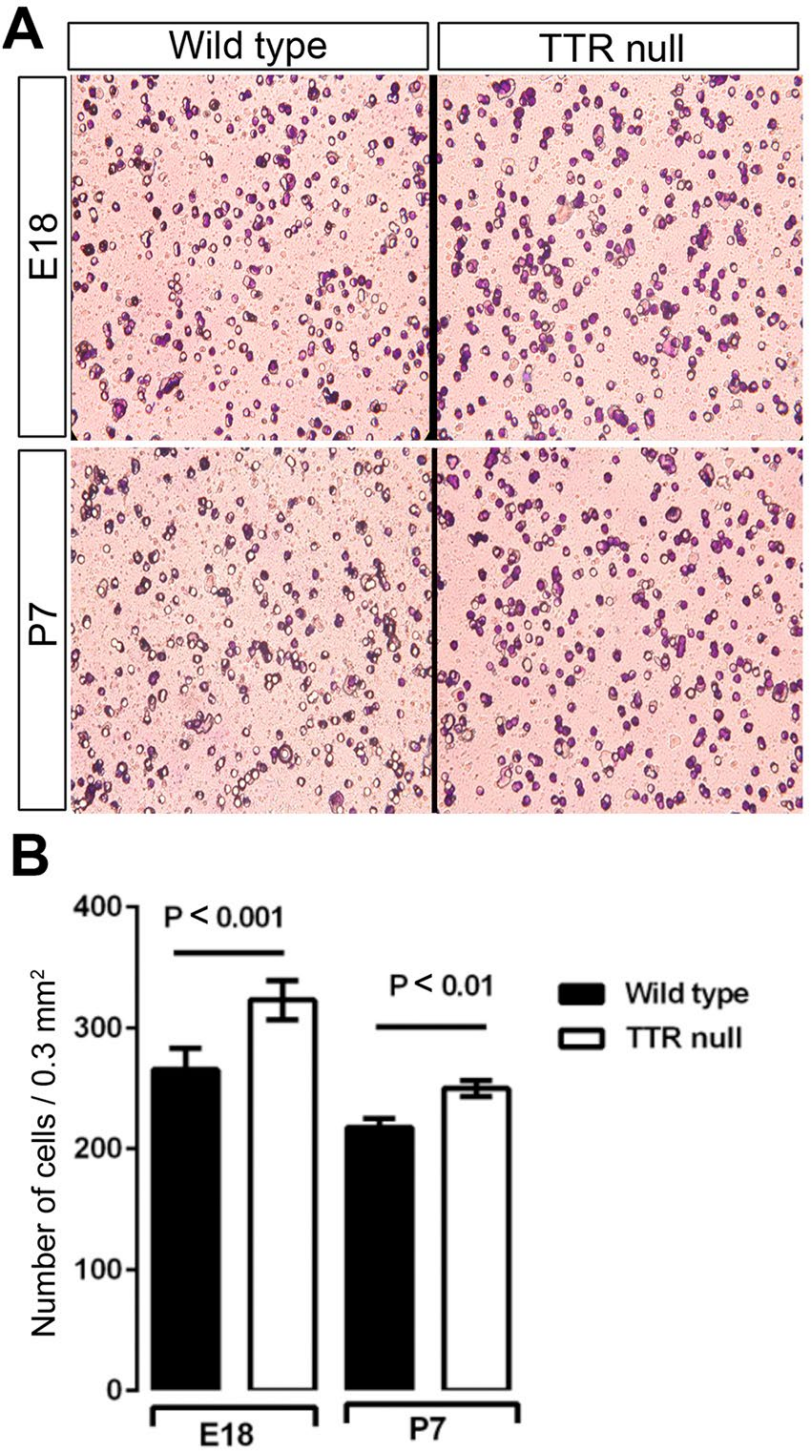
Absence of TTR increases the migration of OPCs isolated from the cerebral cortices of E18 and P7 mice. (**A**) The migratory rates of TTR null OPCs compared to wild type OPCs were analyzed using the modified Boyden chamber assay (migration assay). Representative photomicrograph high power fields (HPF = 0.3 mm^2^) (400X) of migrated OPCs isolated from cortices of E18 and P7 wild type and TTR null mice were stained with crystal violet. (**B**) Data were analyzed from 3 independent experiments for the E18 group and 5 independent experiments for the P7 group. All data were expressed as the mean ± SEM. Statistical comparisons were performed using a one-way ANOVA with post hoc analysis using Sidak’s test as appropriate (*P* < 0.05 was considered statistically significant)^30^.

After having observed the increase in oligodendrocyte numbers within the corpus callosum and anterior commissure of TTR null mice, we posited the question whether the migration rates of OPCs from the developing ventricular zone regions were also altered. To directly address this question, we isolated OPCs from E18 and P7 wild type and TTR null mouse cerebral cortices to perform chemotaxis migration assays. After 12 hours of culture in the migration chamber, the OPCs were stained with cresyl violet and visualized under a x40 objective lens with seven randomly selected fields of the chamber chosen to count the numbers of migrating cells. For each genotype the experiment was repeated 5 times and an overall total of 35 counts were completed (i.e. 7 counts for each chamber) which were then averaged in order to give an estimation of the number of migrating cells (Fig. 6A,B,^30^). The number of migratory OPCs isolated from the cortices of E18 (~ 320 cells/HPF, where each HPF = 0.3 mm^2^) and P7 (~ 250 cells/HPF where each HPF = 0.3 mm^2^) TTR null mice, were significantly higher compared to the number of migratory wild type OPCs (Fig. 6B). The results obtained imply that lack of TTR increased OPC migration.

### Absence of TTR in OPCs decreases the rate of their apoptosis

**Figure 7:**
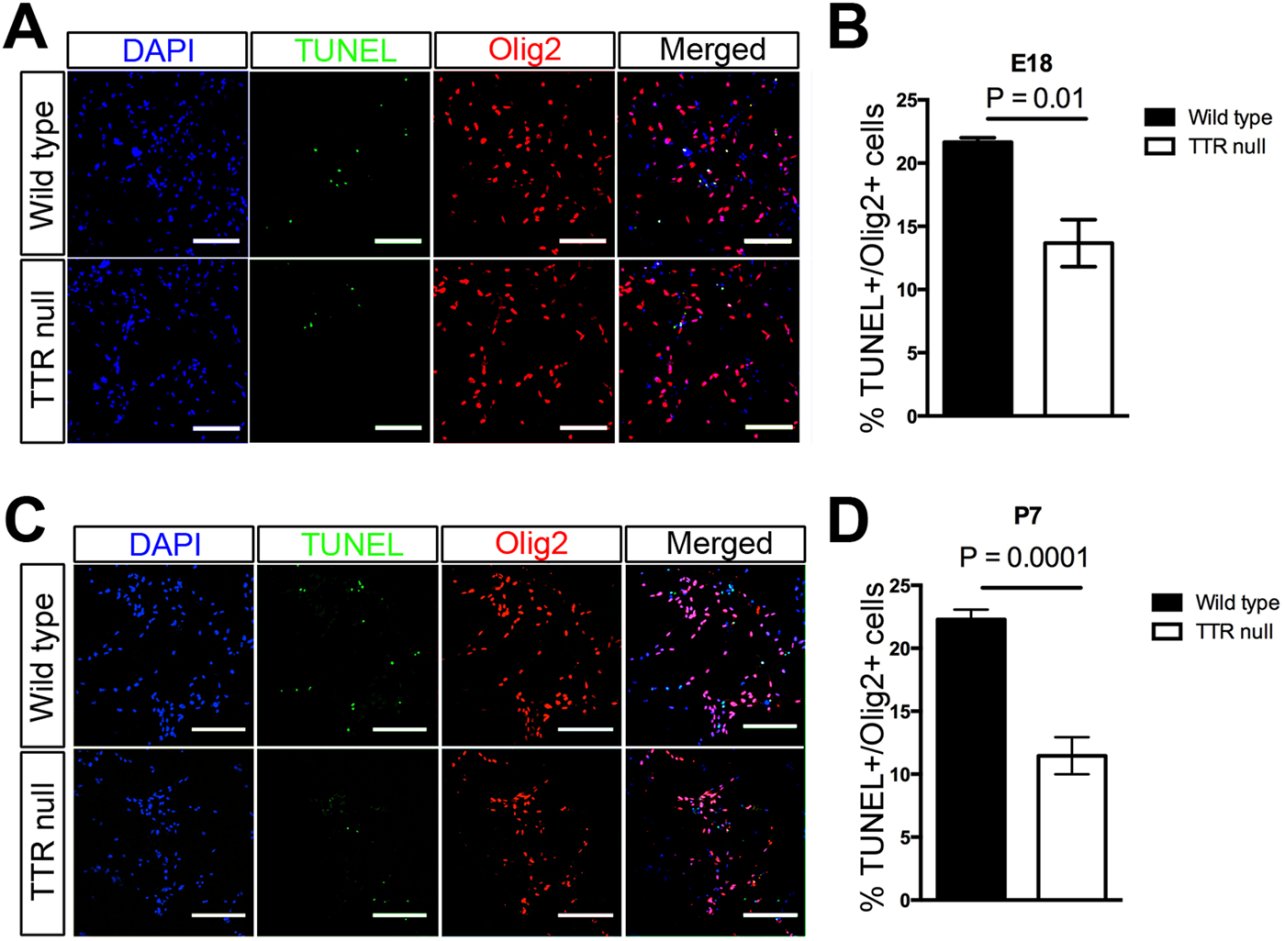
Absence of TTR decreases the rate of apoptosis of OPCs isolated from cerebral cortices of E18 and P7 mice. (**A**) OPCs were isolated from cerebral cortices of E18 TTR null mice. Apoptotic cells were detected via TUNEL (green) staining and oligodendroglial cells were identified by IHC staining for Olig2 (red). Nuclei were stained with DAPI (blue) (scale bar 100 µm). (**B**) Semi-quantitative analysis of OPCs isolated from cortices of E18 wild type and TTR null mice double labeled for TUNEL and Olig2. There was a significant decrease in the number of double-labeled Olig2+ and TUNEL+ OPCs isolated from cortices of E18 TTR null mice compared to those from wild type mice. Data were expressed as the mean ± SEM. Statistical comparisons were performed using Student’s t-test (n = 4 mice per genotype and *P* < 0.05 was considered statistically significant). (**C**) OPCs were isolated from cerebral cortices of P7 mice. Apoptotic cells were detected via TUNEL (green) staining and oligodendroglial cells were identified by IHC staining for Olig2 (red). Nuclei were stained with DAPI (blue) (scale bar 100 µm). (**D**) Semi-quantitative analysis of OPCs isolated from cortices of P7 wild type and TTR null mice double labeled for TUNEL and Olig2. There was a significant decrease in the number of double-labeled Olig2+ and TUNEL+ OPCs isolated from cortices of P7 TTR null mice compared to those from wild type mice. All data were expressed as the mean ± SEM. Statistical comparisons were performed using Student’s *t*-test (n = 5 mice per genotype and *P* < 0.05 was considered statistically significant)^30^.

In order to investigate if TTR has a role in OPC apoptosis, a Terminal-deoxynucleotidyl Transferase Biotin-dUTP Nick End Labeling (TUNEL) assay was performed on OPCs isolated from cortices of E18 and P7 mice and then proliferated for one week in culture. The incorporated biotinylated dUTP was labeled with Alexa Flour 488 and double labeled for the Olig2 marker (Fig. 7A,C, respectively,^30^). Quantitation of the data from four experiments showed a clear decrease in the population of OPCs positive for TUNEL following their isolation from the cortices of E18 and P7 TTR null mice compared to those from wild type mice (Fig. 7B,D). These results suggest a role for TTR in promoting the frequency of developmentally regulated apoptosis of OPCs.

### Optic nerves of adult TTR null mice display higher concentrations of phosphorylated AKT compared to wild type mice

To identify downstream signalling involved during developmental myelination in TTR null and wild type mice, we focused our analysis on the active kinases AKT, ERK1/2, that have been previously defined in oligodendrocyte differentiation, proliferation and cell survival^37–40^. The levels of the active phosphoproteins were quantified by western blotting and immunoprecipitation from lysed optic nerves of adult wild type and TTR null mice (n = 9–10 mice per genotype; 18–20 optic nerves).

**Figure 8:**
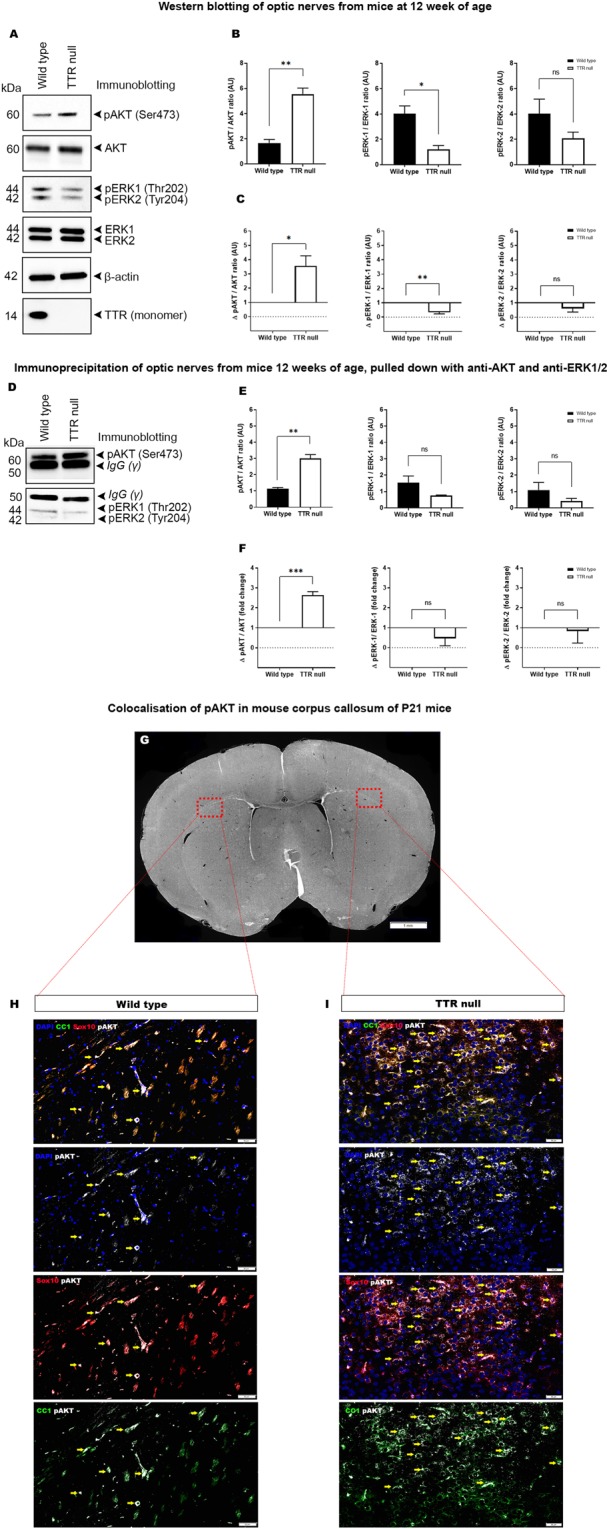
Optic nerves of adult TTR null mice display higher concentrations of phosphorylated AKT compared to wild type mice. (**A**) Western blot analysis of total protein lysate (5 µg) from optic nerves of wild type and TTR null mice (n = 18–20 optic nerves from 9–10 mice per genotype), identifying bands of corresponding molecular weights for proteins: pAKT, AKT, pERK1, pERK2, ERK1, ERK2, β-actin and TTR. (**B**) Densitometric analysis of immunoblots (n = 3) comparing wild type and TTR null mice displayed as a phosphorylated to non-phosphorylated protein ratio for AKT and ERK1, ERK2, normalised against β-actin. (**C**) Densitometric analysis of immunoblots (n = 3) from comparing the fold change of TTR null to wild type mice of phosphorylated to non-phosphorylated AKT, ERK1 and ERK2. (**D**) Immunoprecipitation and Western blotting of AKT, ERK1 and ERK2 from total protein lysates (100 µg) (n = 18–20 optic nerves from 9–10 mice per genotype). (**E**) Densitometric analysis of immunoblots (n = 3) following immunoprecipitation of anti-AKT, anti-ERK1 and anti-ERK2 directly comparing wild type and TTR null mice displayed as a phosphorylated to non-phosphorylated protein ratio, normalised against β-actin. (**F**) Densitometric analysis of immunoblots (n = 3) following immunoprecipitation comparing the fold change of TTR null to wild type mice of phosphorylated to non-phosphorylated AKT, ERK1 and ERK2. (**G**) Regions of the lateral corpus callosum in wild type and TTR null mice at ages P21 selected for image analysis to identify localisation of pAKT in mature oligodendrocytes (10 µm formalin fixed paraffin embedded sections, scale bar 1 mm). (**H**) Immunofluorescent images obtained from the lateral corpus callosum of P21 wild type null mice displaying colocalisation of pAKT around the nuclei of Sox10- CC1-positive oligodendrocytes (yellow arrows). Panels top to bottom: DAPI, CC1, Sox10, pAKT merge; DAPI, pAKT; Sox10, pAKT; CC1, pAKT (scale bar 50 µm). (**I**) Immunofluorescent images obtained from the lateral corpus callosum of P21 TTR null mice displaying increased colocalisation of pAKT around the nuclei of Sox10- CC1-positive oligodendrocytes (yellow arrows). Panels top to bottom: DAPI, CC1, Sox10, pAKT merge; DAPI, pAKT; Sox10, pAKT; CC1, pAKT (scale bar 50 µm).

Densitometric analyses (Fig. 8B,C) of western immunoblots (n = 3) (Fig. 8A) for optic nerve protein lysates from wild type and TTR null mice were performed using antibodies against total and phosphorylated AKT and ERK1/2 (pAKT and pERK1/2), normalised against β-actin. We demonstrated that the optic nerves of TTR lysates exhibited elevated pAKT levels when normalized to total AKT which was estimated to be ~ 2.6-fold change to that observed in wild type mice (Fig. 8B–F, *P* < 0.01). However, the optic nerve lysates of TTR null mice demonstrate a significant reduction in pERK-1 (~0.6-fold) when compared to wild type mice (Fig. 8B–F).

When we interrogated the neural cell types responsible for the elevated pAKT levels expressed in the CNS of TTR null, compared with that exhibited in wild type mice we could identify that the localization of expression was predominantly occurring on CC1- and SOX10-positve oligodendroglial lineage cells (Fig. 8I). These data collectively support the hypothesis that TTR null mice exhibit potentiated AKT phosphorylation in oligodendroglial lineage cells. Whether this specific signaling can drive increased numbers and maturation of oligodendrocytes, thereby promoting hypermyelination in the CNS of TTR null mice remains to be elucidated.

### TTR reduces proliferation and migration of OPCs *in vivo*

**Figure 9:**
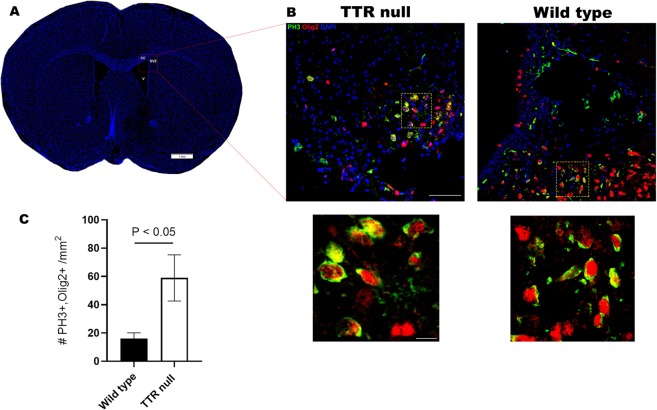
TTR reduces proliferation and migration of OPCs *in vivo*. (**A**) Coronal section mouse brain labelled for DAPI-positive nuclei indicating boxed area of interest within the corpus callosum (CC) and subventricular zone (SVZ) where representative images were taken and cell densities calculated for semi-quantitative analyses of Olig2- and PH3-positive cells, from wild type and TTR null mice, respectively (scale bar 1 mm). (**B**) Representative confocal images of coronal brain cryosections (12 µm) between bregma 0.6 to 1.3 representing cells stained for Olig2 (red), PH3 (green) and DAPI (blue) in P4 TTR null (left) and wild type mice (right) (scale bar 50 µm). Region of individual cells magnified (yellow box) and illustrated below (scale bar 5 µm). **(C)** A semi-quantitative cell density analysis of double positive PH3/Olig2 cells counted per mm^2^ from P4 wild type and TTR null mice. Data were expressed as the mean ± SEM (n = 5–6 per genotype). Mouse genotypes were compared using an unpaired Student’s *t*-test (*P* < 0.05 was considered statistically significant).

To investigate the *in vivo* relevance of TTR in regulating proliferating oligodendroglial cells (as observed in culture from the isolated progenitor cells), regions of corpus callosum (CC) and (SVZ) of wild type and TTR null mice were histologically analyzed (n = 5 or 6 mice per group). We demonstrated that Olig2- and PH3- double-labeled cells were prevalent in areas of the SVZ and the CC in both wild type and TTR null samples at the early postnatal age of P4 (Fig. 9A,B). Semi-quantitation of the Olig2- and phospho-histone H3- double-labeled cells, demonstrated that the mean densities of PH3/Olig2 cells per mm^2^ of brain tissue area, were 16.11 ± 4.04 for wild type and 58.97 ± 16.33 for TTR null samples, respectively. This difference represented a 3.7-fold increase in proliferating oligodendrocytes in TTR null compared to wild type mice (*P* = 0.045) (Fig. 9C).

## Discussion

THs are well known to be integral to brain development in vertebrates^41^. In eutherian mammals, this is regulated through the contributions from the maternal circulation and the fetal thyroid gland after it becomes active during gestation. The distribution of circulating THs around the body via the bloodstream is achieved by binding to TH distributor proteins: albumin, TTR and thyroxine-binding globulin. In humans, more than 99% of THs in blood is bound to TH distributor proteins. Of these, TTR is responsible for the bulk of THs being bioavailable to tissues (for discussion, see^15^). Of the three TH distributor proteins, TTR is the only one to be synthesized in the CNS. One site of TTR synthesis in the CNS is the epithelial cells of the choroid plexus. This TTR is involved in moving thyroxine (T4) from the blood into the CSF^6–9^. Despite these well-characterized roles of TTR, until now, no data have suggested a role for TTR in developing oligodendrocytes and myelination. In this study, we identified a hypermyelination phenotype in the brains of TTR null mice during postnatal development. The hypermyelination phenotype coincided with substantial increases in the density of oligodendrocytes in the medial area of the corpus callosum and in the anterior commissure during development, starting from P7 and evident until 12 weeks of age. The biological relevance of these findings may be that as a consequence of the absence of TTR, the derivation of maturing oligodendrocytes from NSC populations, at least within the SVZ, is enhanced. The first *in vitro* sign of that alteration was the significant increase in the proliferation rate of NSC neurosphere colonies isolated from the SVZ of P21 TTR null mice. Although further confirmation is required, our data describes for the first time that TTR may be acting as a repressor of NSC and OPC proliferation. In particular, an abrogation of TTR in OPCs may lift this repression, thereby promoting the proliferation and maturation toward a myelinating phenotype.

Telencephalic oligodendrocytes are derived from progenitors that migrate from the medial ganglionic eminence and the anterior entopeduncular area^42,43^. In the adult mammalian brain, mature myelinating oligodendrocytes are constantly produced from local OPCs situated in the brain parenchyma^44,45^ and from precursor cells residing in the SVZ^46,47^. The influence of THs upon oligodendrocyte differentiation is regulated at the molecular transcriptional level, by which myelin genes such as myelin basic protein, myelin associated glycoprotein and cyclic nucleotide phosphatase are up-regulated once the nuclear hormone receptors bind to the TH response elements in their gene promoter regions^48–51^. However, timing in the maturation of oligodendrocytes may indeed require the effects of TH that are governed by cell cycle check points^52–54^. Furthermore, the effect of specifically removing T3 in the SVZ, has been proposed to regulate glial fate determination which favours OPCs rather than neuronal cells^19^. Despite these potent effects upon oligodendrogenesis, the requirement for TH systemic distribution remains a critical physiological mechanism that may also contribute substantially *in vivo* for oligodendrocyte maturation and myelination. Our observation that the absence of TTR promoted differentiation of NSCs into the glial, at the expense of the neuronal lineage, underpins the importance of this particular TH distributor protein in the early neural cell specification stage. This was further supported by our data that illustrated enhanced proliferation and mature oligodendrocyte numbers within the major commissures of the CNS, and as a corollary, a substantial decrease in the number of apoptotic OPCs. These data may suggest that indeed TTR plays an active role in determining the cell fate of neural precursors, in particular by limiting the derivation of maturing OPCs toward myelinating oligodendrocytes. However, the precise mechanism by which TTR exerts this influence upon OPCs is yet to be defined.

Whether the increase in OPC maturation observed in the TTR null mice associated with a reduction in oligodendrocyte apoptosis, resulted in the hypermyelination phenotype, is yet to be defined in a fate-map analysis. However, we have previously identified that the progenitor cells in the SVZ display enhanced cell viability in TTR null mice^18^ suggesting a direct link of TTR with the apoptotic pathway. Recently, an elegant study in C. *elegans* demonstrated a direct interaction of *ttr-52* (the ortholog of mammalian *ttr*) with phosphatidyl serine (PS) to tag areas of apoptotic clearance in transected axons by phagocytes for axonal reconnection and fusion^55^. This mechanism was shown to be elicited through the PS receptor apoptotic pathway. Of great importance to the current study was the previously reported effect of TTR-52 as a secreted protein in the regulation of embryonic cell apoptosis of exoplasmic leaflet PS-labelled cells^56^. Indeed, these investigators suggested that the mechanism by which apoptotic cells are cleared is through a signalling pathway that involves TTR-52 promoting the efflux of PS in extracellular vesicles. It is therefore tantalizing to posit that the reduction in apoptotic cells in the SVZ as well as the reduced levels of OPC death observed in the TTR null mice may be a direct consequence of a reduced capacity to tag these cells with TTR bound to PS expressed on the exoplasmic leaflet, thereby limiting their clearance from the developing brain. However, this hypothesis would require direct evidence for its demonstration in OPCs.

However, there exists a complexity concerning oligodendrogenesis and myelination that can include various growth factors, hormones and signalling molecules that play key but disparate roles in controlling OPC proliferation and survival. Here, we report that TTR could be a factor with direct or indirect roles in controlling the cell cycle and exerting OPC proliferation and survival repression effects. The first possible mechanism for TTR’s influence on the cell cycle is via THs, which influence the OPC cell cycle^20^. Compared to other TH distributor proteins (albumin and thyroxine-binding globulin), TTR has an intermediate affinity for T3 and T4^57^. Therefore, it is possible that endogenous TTR, shown to be synthesized by OPCs, may regulate the transcription of TH-controlled genes. Indeed, a recent study observed a decrease in neurosphere formation and a decrease in cell proliferation after the provision of exogenous TTR to neurosphere cell cultures implicating TTR in the transcriptional regulation of cell cycle events^13^. However, the specific role of TTR in oligodendrocyte maturation may be unrelated to its interaction with TH and further experiments are required to address these key questions.

Oligodendroglial cell proliferation, migration, survival and differentiation rely on the coordinated transduction of phosphatidylinositol-3-phosphate kinase (PI3K)/AKT and extracellular signal-regulated protein kinases 1 and 2 (ERK1/ERK2), regulate processes essential to CNS myelination^58,59^. Studies have demonstrated that myelin thickness is increased in transgenic mice with the overexpression of the PI3K/AKT pathway^60^. Furthermore, conditional gene knockout mouse models that disrupt the expression of Erk1/2 genes have a reported reduction of myelin thickness and an overall degradation of axonal integrity from ERK1/2 lack of function in oligodendrocytes^38^. Here, we report an increased expression of AKT protein in the optic nerves of TTR null mice, which implicates a regulatory effect imposed on oligodendrocyte maturation and myelin formation governed by TTR expression. Our observation that TTR null mice demonstrated increased pAKT expression levels in optic nerves and localised to oligodendrocytes of the corpus callosum, is indicative of removal in signal repression governing oligodendroglial maturation. Key mechanisms that control oligodendrogenesis are now being uncovered and include the time-dependent PI3K/Akt intracellular signalling to drive myelination through downstream mammalian target of rapamycin complex 1-specific mRNA translation and lipid biosynthesis^61^). How TTR regulates this signaling cascade in oligodendrocytes is of critical importance to understanding its repressive effects upon maturation and myelination, warranting thorough investigation with direct implications to demyelinating diseases.

Our current study has demonstrated that TTR null mice have a hypermyelination phenotype in the commissural fibres of their brains. This specific hypermyelination phenotype is regulated by accelerated oligodendrocyte maturation (proliferation and migration) and density, as a consequence of the absence of TTR synthesis by OPCs. This suggests that TTR may have a role in oligodendrocyte development and in the process of myelination. This could have implications for future strategies for the development of therapeutics for diseases of demyelination such as multiple sclerosis. Further studies are underway to identify the mechanisms of TTR action in OPC biology.

## Supplementary information

Supplementary information.

## Data Availability

Original data are available upon request.
