# Supplementary material for: The Role of Transthyretin in Oligodendrocyte Development
Source: Sci Rep. 2020 Mar 6;10:4189. doi: 10.1038/s41598-020-60699-8 (PMC7060235; doi:10.1038/s41598-020-60699-8)

Supplementary data

The Role of Transthyretin in Oligodendrocyte Development

Bandar Alshehri, Maurice Pagnin, Jae Young Lee, Steven Petratos and Samantha J. Richardson

Supplementary FIGURE 1: TTR is synthesized in proliferating and differentiating OPCs.

To determine if OPCs in proliferating and maturing cell lines synthesise TTR, human OPCs derived from NIH approved H9 human embryonic stem cells were analysed. **(A)** TTR mRNA was detected in proliferating and in differentiating OPCs derived from NIH approved H9 hESCs. GAPDH cDNA was used as the internal control. Olig2 cDNA was used as the oligodendroglial cell marker. TTR cDNA was detected in both proliferating and differentiating oligodendroglial cells. The DNA template was omitted in negative control reactions. The relative ratio of TTR cDNA bands was calculated and normalized to the GAPDH bands.

(B) Western blot analysis revealing TTR in OPCs derived from NIH approved H9 hESCs. Left panel: TTR in protein extracted from OPCs cultured in proliferation media. Right panel: TTR in protein extracted from OPCs cultured in differentiation media. Human albumin was used as the negative control and human TTR was used as the positive control for both experiments.

(C) Western blot analysis of media collected from oligodendroglial cells that had been proliferating (left panel) or differentiating (right panel). Media had been concentrated 20-fold prior to western analysis. TTR was not detected in the concentrated media.

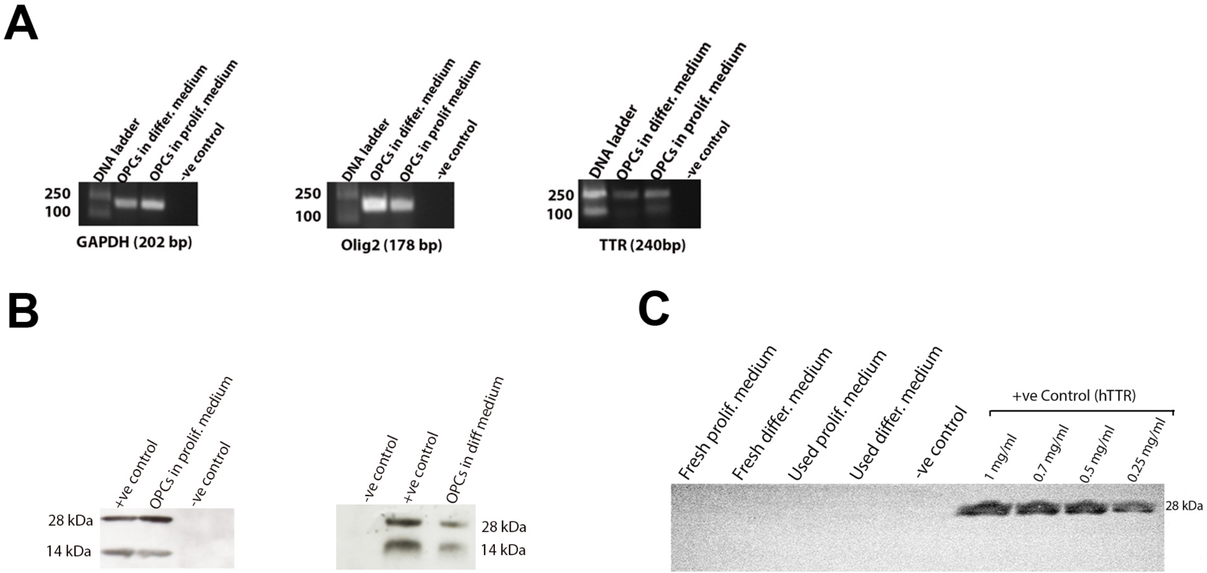
A
DNA ladder
OPCs in differ. medium
OPCs in prolif. medium
-ve control
250
100
GAPDH (202 bp)
Olig2 (178 bp)
TTR (240bp)
B
+ve control
OPCs in prolif. medium
-ve control
28 kDa
14 kDa
OPCs in diff medium
C
Fresh prolif. medium
Fresh differ. medium
Used prolif. medium
Used differ. medium
-ve control
+ve Control (hTTR)
1 mg/ml
0.7 mg/ml
0.5 mg/ml
0.25 mg/ml
28 kDa

Supplementary figures

The Role of Transthyretin in Oligodendrocyte Development

Bandar Alshehri, Maurice Pagnin, Jae Young Lee, Steven Petratos, Samantha J. Richardson.

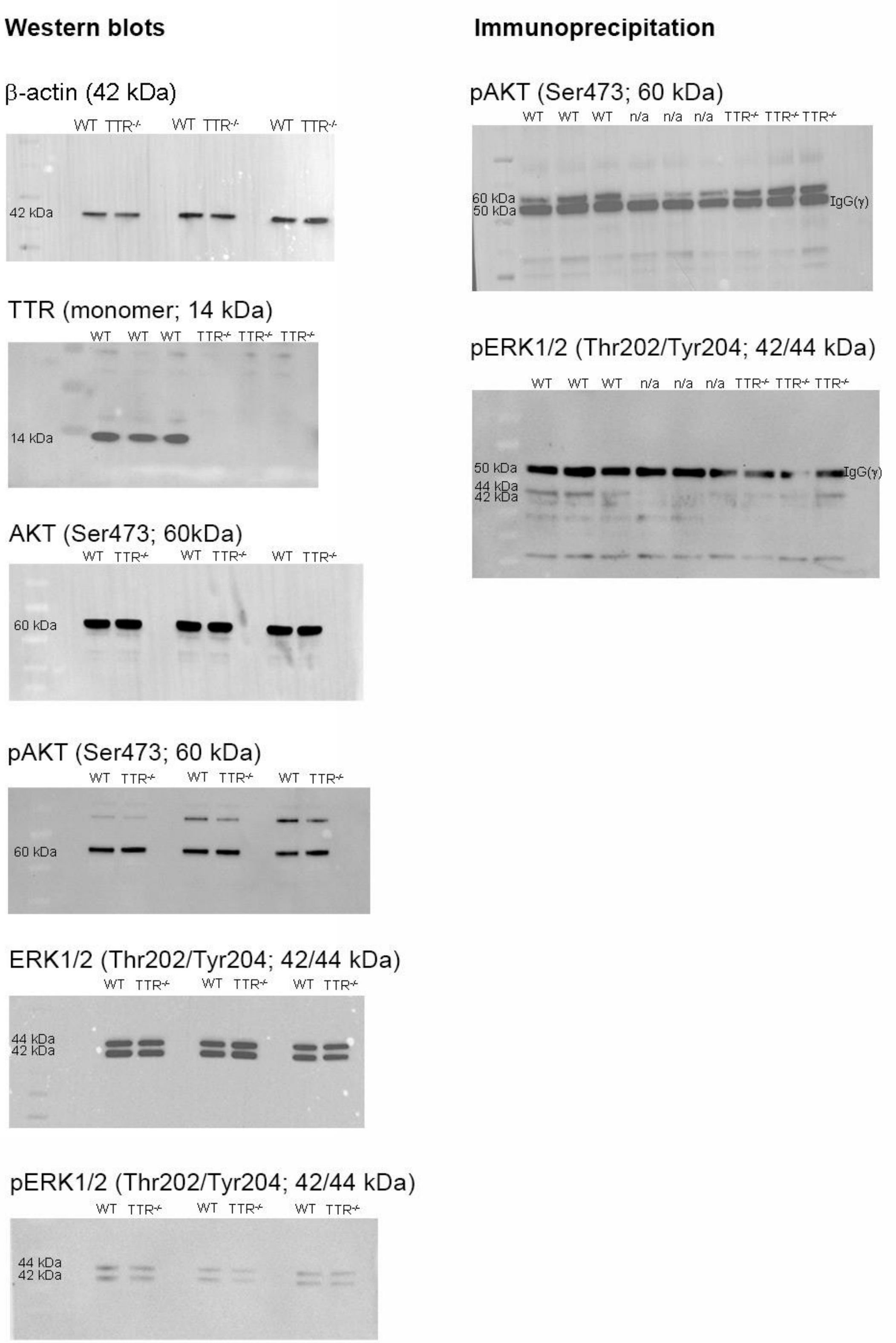

Supplement: Supplementary file 1 — Supplementary information. [file 41598_2020_60699_MOESM1_ESM.pdf]
